# ModuleDiscoverer: Identification of regulatory modules in protein-protein interaction networks

**DOI:** 10.1038/s41598-017-18370-2

**Published:** 2018-01-11

**Authors:** Sebastian Vlaic, Theresia Conrad, Christian Tokarski-Schnelle, Mika Gustafsson, Uta Dahmen, Reinhard Guthke, Stefan Schuster

**Affiliations:** 1 Leibniz Institute for Natural Product Research and Infection Biology - Hans-Knöll-Institute, Systems Biology and Bioinformatics, Jena, 07745 Germany; 2 Friedrich-Schiller-University, Department of Bioinformatics, Jena, 07743 Germany; 3 University Hospital Jena, Friedrich-Schiller-University, General, Visceral and Vascular Surgery, Jena, 07749 Germany; 4 Linköping University, Bioinformatics, Department of Physics, Chemistry and Biology, Linköping, 581 83 Sweden

## Abstract

The identification of disease-associated modules based on protein-protein interaction networks (PPINs) and gene expression data has provided new insights into the mechanistic nature of diverse diseases. However, their identification is hampered by the detection of protein communities within large-scale, whole-genome PPINs. A presented successful strategy detects a PPIN’s community structure based on the maximal clique enumeration problem (MCE), which is a non-deterministic polynomial time-hard problem. This renders the approach computationally challenging for large PPINs implying the need for new strategies. We present ModuleDiscoverer, a novel approach for the identification of regulatory modules from PPINs and gene expression data. Following the MCE-based approach, ModuleDiscoverer uses a randomization heuristic-based approximation of the community structure. Given a PPIN of *Rattus norvegicus* and public gene expression data, we identify the regulatory module underlying a rodent model of non-alcoholic steatohepatitis (NASH), a severe form of non-alcoholic fatty liver disease (NAFLD). The module is validated using single-nucleotide polymorphism (SNP) data from independent genome-wide association studies and gene enrichment tests. Based on gene enrichment tests, we find that ModuleDiscoverer performs comparably to three existing module-detecting algorithms. However, only our NASH-module is significantly enriched with genes linked to NAFLD-associated SNPs. ModuleDiscoverer is available at http://www.hki-jena.de/index.php/0/2/490 (Others/ModuleDiscoverer).

## Introduction

Structural analysis of intracellular molecular networks has attracted ample interest over several decades^1^. This includes cellular networks such as protein interaction maps^2^, metabolic networks^3,4^ transcriptional regulation maps^5^, signal transduction networks^6,7^ as well as functional association networks^8^. Recent advances in the field of network medicine have focused on the identification of disease-associated modules within the organism-specific interactome^9^. The interactome captures interactions between all molecules of a cell^10^ and is represented by a graph composed of nodes denoting cellular molecules that are connected by edges representing interactions between them. Within the interactome, modules are sub-graphs that can be linked to phenotypes such as diseases or traits. Up to date, the identification of disease-associated modules has been applied mostly based on protein-protein interaction networks (PPINs) of *Homo sapiens*. They have been successfully identified for, e.g., asthma^11^, inflammatory and malignant diseases^12^, obesity and type-2-diabetes (among others)^13^ as well as different subtypes of breast cancer^14–16^, providing new in-depth insights into the underlying molecular mechanisms of the respective disease. For example, biomarker identification for the classification of 402 breast tumor samples into their respective subtype was successfully performed based on subtype-specific protein signaling networks^15^. Furthermore, the same study highlighted that strongly connected genes (i.e., hub genes) present in either subtype-specific network are valid drug targets for the respective subtype.

**Figure 1:**
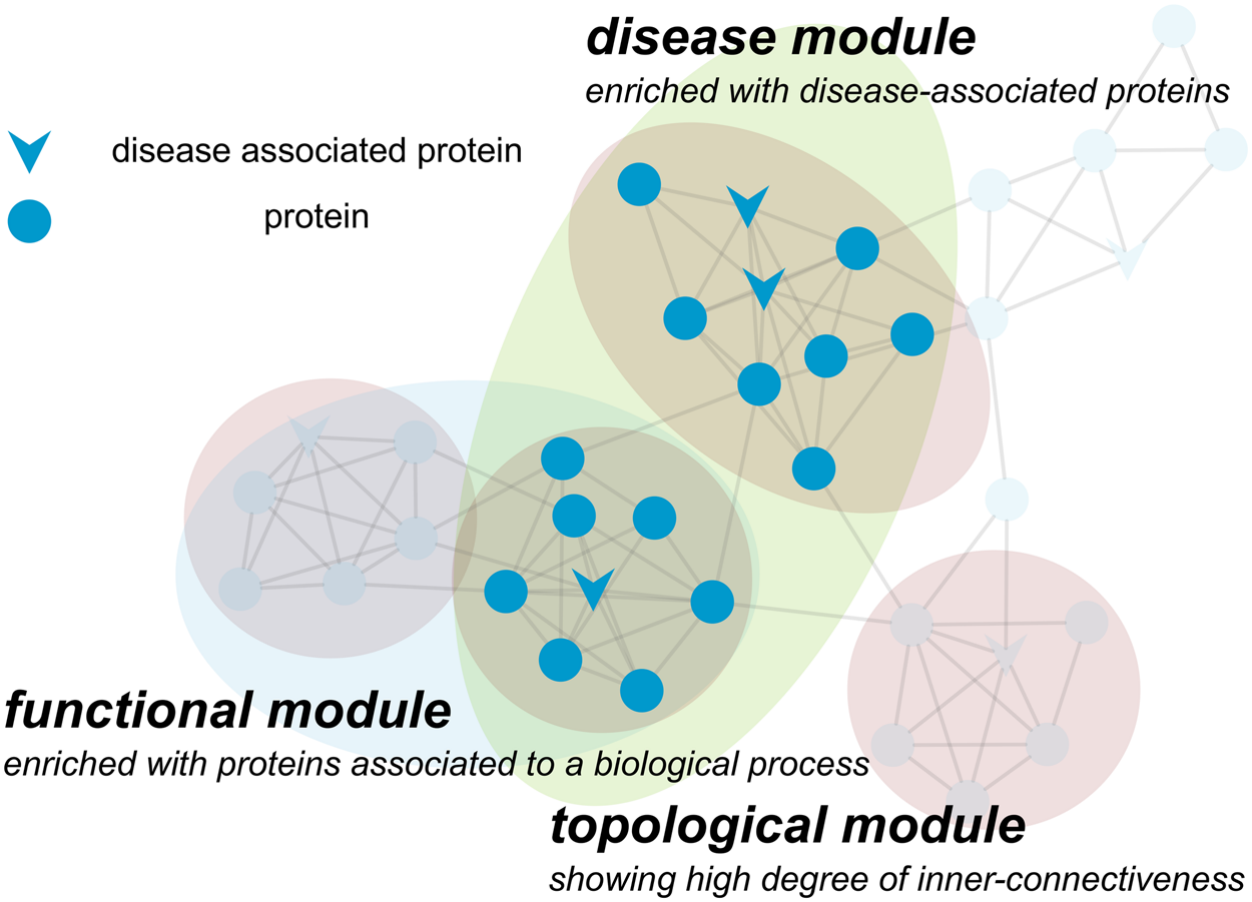
The concept of disease modules exemplified using a sample PPIN. One or more topological modules (highlighted red) contain proteins involved in similar biological processes forming functional modules (highlighted blue). A disease module (highlighted green) is a sub-network of proteins enriched with disease-relevant proteins, e.g., known disease-associated proteins.

There are three fundamental assumptions underlying the identification of disease modules^17^ (Fig. 1). Firstly, entities forming dense clusters within the interactome (topological modules) are involved in similar biological functions (functional modules). Secondly, molecules associated to the same disease, such as disease-associated proteins, tend to be located in close proximity within the network, which defines the disease module. Thirdly, disease modules and functional modules overlap. Thus, a disease relates to the breakdown of one or more connected functional modules.

A variety of approaches have been presented specifically for the identification of disease modules. They can be categorized into two different groups. On the one hand, there are algorithms that make use of known disease-associated molecules or genetic loci, the known interactome as well as some association function for the identification of disease modules and/or new disease-associated molecules^18–22^. For example, the disease module detection (DIAMOnD) algorithm^20^ utilizes known disease-associated proteins (seed proteins) to identify proteins (DIAMOnD proteins) significantly connected to seed proteins. Iterative application of the algorithm results in a growing disease module with a ranked list of DIAMOnD proteins, i.e., candidate disease-associated proteins. On the other hand, there are algorithms that identify disease modules as well as disease-associated molecules *‘ab initio’* based on the projection of omics data onto the interactome in conjunction with a community structure detecting algorithm^12,13,23^. Like topological modules, communities are groups of proteins with higher within-edge density compared to the edge density connecting them^24^. For example, the approach presented by Barrenäs *et al*.^13^ identifies protein communities by decomposition of the human PPIN into sub-graphs of maximal cliques. A clique is a sub-graph of the PPIN, where each pair of proteins is connected by an edge. A maximal clique is a clique that is not part of a larger clique. The regulatory module is then formed by the union of all maximal cliques that are significantly enriched with disease-associated-proteins, e.g., differentially expressed genes.

The idea of disease modules can obviously be generalized towards the detection of regulatory modules underlying an arbitrary phenotype of any organism. This can be of high interest, e.g., for the molecular characterization of animal models of diverse human diseases. This includes animal models of infectious diseases such as fungal infections with *Candida albicans* and *Aspergillus fumigatus* ^25^, animal models of inflammation^26^, asthma^27^ as well as metabolic diseases such as fatty liver disease (FLD)^28^. Since animal models reflect only certain aspects of the human disease phenotype^29^, identification of the underlying regulatory module can provide additional information regarding the functional context in which such models are valid. A variety of algorithms for the identification of such phenotype (or condition)-specific modules in PPINs have been published^30^. Like the MCE-based approach by Barrenäs *et al*.^13^, so called ‘module cover approaches’ (see Batra *et al*.^31^) such as MATISSE^32^, DEGAS^33^ and KeyPathwayMiner^34^ consider the detection of differential gene expression as a separate pre-processing step and can handle proteins in the PPIN with missing expression information. In contrast to the MCE-based approach, these algorithms avoid assumptions about the community structure. In turn, they introduce additional parameters controlling, e.g., the allowed noise in the network structure (DEGAS and KeyPathwayMiner) or the module size (MATISSE), or introduce additional assumptions such as the expected fraction of similarly expressed genes in the regulatory module (MATISSE). The optimization problem underlying these approaches is non-deterministic polynomial time (NP)-hard (see Batra *et al*.^31^, Ulitsky *et al*.^32^ and Eblen *et al*.^35^, respectively). Thus, application of any of these algorithms to large-scale PPINs becomes computationally challenging. While heuristics were presented for DEGAS, KeyPathwayMiner and MATISSE, an efficient heuristic following the idea of the MCE-based approach is missing.

We present ModuleDiscoverer, a new approach to the *ab initio* identification of regulatory modules. ModuleDiscoverer is a heuristic that, based on the idea of the MCE-based approach, approximates the PPIN’s underlying community structure by iterative enumeration of cliques starting from random seed proteins in the network. We identify the regulatory module underlying a diet-induced rat model of non-alcoholic steatohepatits (NASH), the severe form of the non-alcoholic fatty liver disease (NAFLD). The identified NASH-regulatory module is then validated using NAFLD-associated single nucleotide polymorphism (SNP) data from independent genome-wide association studies (GWASs) as well as gene enrichment tests based on known gene-to-disease relations. We compare our results to those derived from DEGAS, MATISSE and KeyPathwayMiner. Finally, we show that our NASH-module reflects histological and clinical parameters as reported by Baumgardner *et al*.^36^, who first introduced the animal model.

## Results

### ModuleDiscoverer: detection of regulatory modules

**Figure 2:**
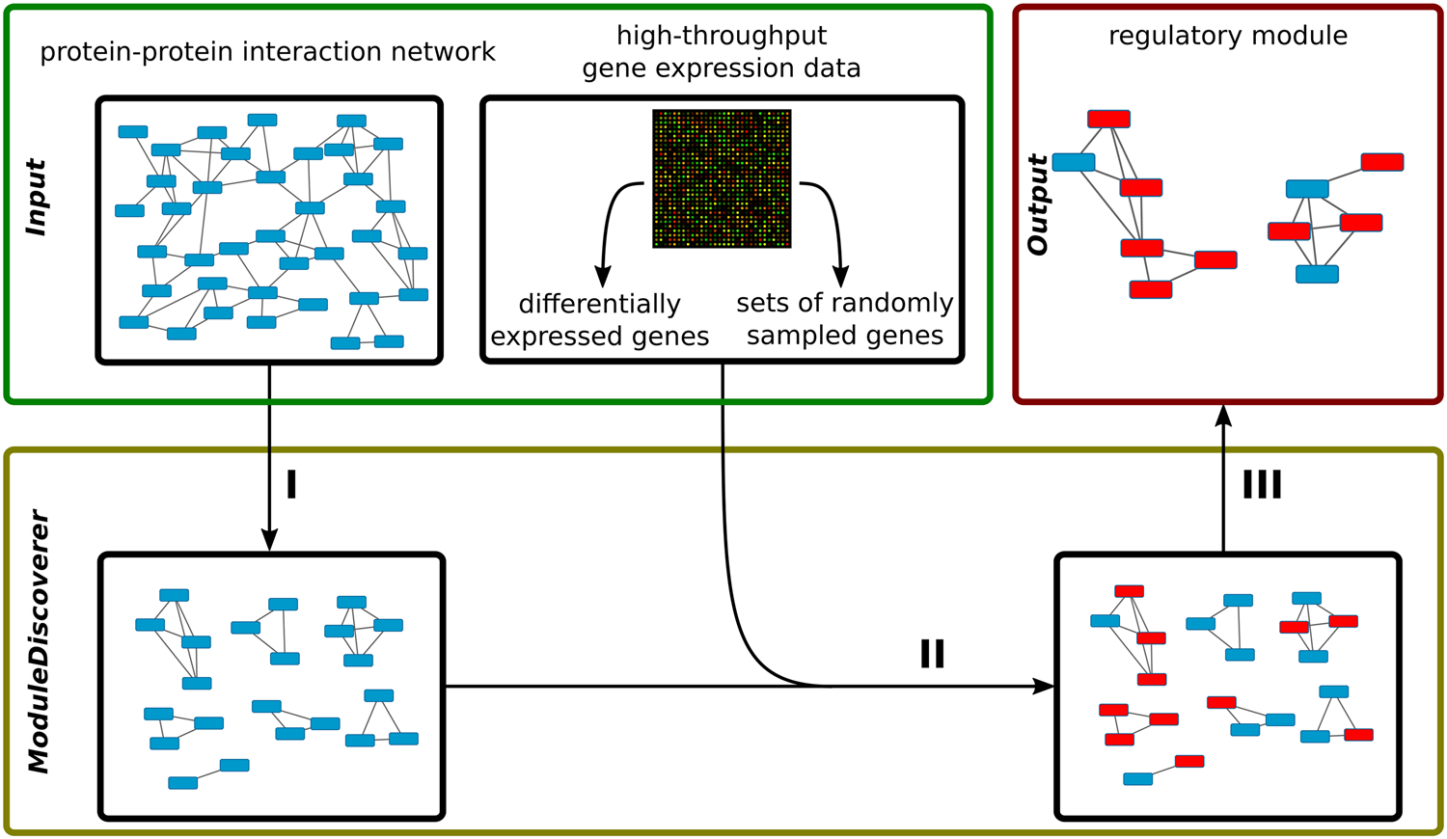
Given a PPIN and gene expression data (Input), the algorithm works in three steps. Step I) The community structure underlying the PPIN is approximated by the identification of protein cliques. Step II) Identification of cliques significantly enriched with DEGs. Step III) Assembly of the regulatory module based on the union of significantly enriched cliques.

The detection of regulatory modules is divided into three steps I–III (Fig. 2). Starting with a PPIN (Fig. 2, Input) the algorithm first approximates the underlying community structure by iterative enumeration of protein cliques from random seed proteins in the network (Fig. 2, I). Next, DEGs obtained from high-throughput gene expression data in conjunction with sets of randomly sampled genes (Fig. 2, Input) are used to calculate a p-value for each clique (Fig. 2,II). Finally, significantly enriched cliques are assembled (Fig. 2,III) resulting in the identified regulatory module (Fig. 2, Output).

#### Step I: Approximation of the PPIN’s community structure

**Figure 3:**
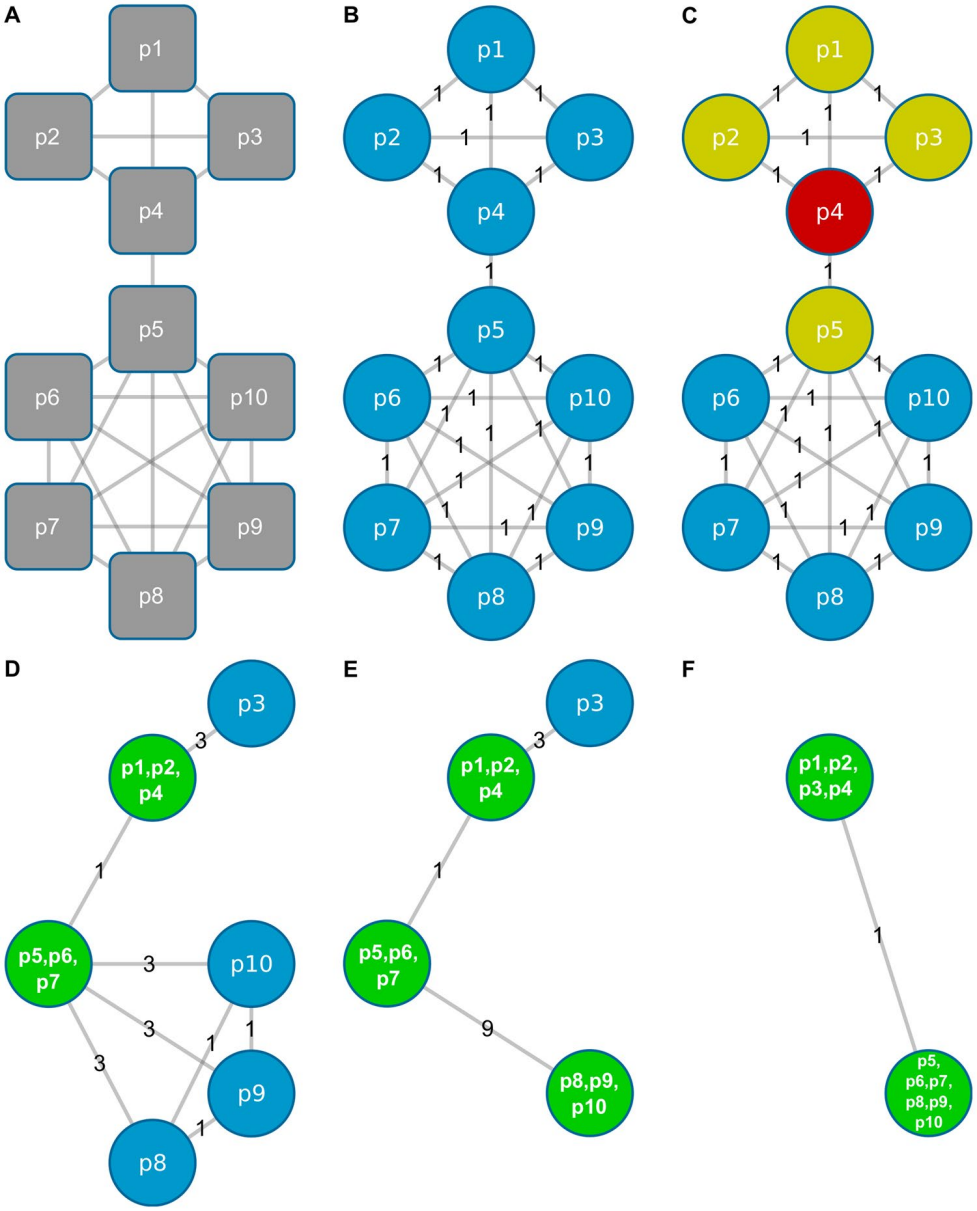
Clique enumeration using ModuleDiscoverer. (**A**) Sample PPIN with 10 proteins and 26 known relations. (**B**) Representation of the PPIN as an undirected labeled graph with each vertex representing one of the proteins in (**A**). The edge weight denotes for the number of existing relations between its connecting nodes. (**C**–**F**) Red vertices denote for seed nodes. Yellow vertices are first neighbors of seed nodes. Green vertices represent cliques. Their label represents clique forming proteins.

Approximation of the community structure underlying the PPIN (Fig. 2, I) is composed of three phases: transformation, identification and extension. In brief, the PPIN is transformed into a graph with labeled nodes and edges (Fig. 3A,B). Starting from one or more random seed nodes, the algorithm then identifies minimal cliques of size three (Fig. 3C,E). Finally, all minimal cliques are stepwise extended competing for nodes in the network until no clique can be extended further (Fig. 3F).

The number of seed nodes used defines two strategies for the enumeration of cliques, the single-seed and the multi-seed approach. Notably, there are advantages as well as disadvantages for both strategies (Supplementary File S1). The single-seed approach identifies cliques using only one seed node in the PPIN. This is suitable for the identification of regulatory modules that are comparable to the results of current, MCE-based algorithms. However, in dense regions of highly overlapping cliques, the single-seed approach favors the enumeration of large maximal cliques. Consequently, proteins that are part of only small cliques can be missed. In contrast, the use of two or more seed nodes (the multi-seed approach), which compete for nodes during the enumeration of cliques, leads to a breakdown of large maximal cliques. While this increases the probability for proteins contained in small cliques only to become part of the final regulatory module, it also leads to an inflation of the regulatory module with proteins not associated to DEGs. Concluding, the multi-seed based regulatory modules can be seen as a comprehensive extension to the single-seed based regulatory modules. In the following example we will illustrate our approach showing one iteration of ModuleDiscoverer using three seed proteins (*p*4, *p*6 and *p*9).

Phase 1 of Step I: Transformation of the PPIN into a labeled graph: Figure 3(A) shows a PPIN as provided by databases such as STRING^37^. It consists of 10 nodes representing the proteins *p*1 to *p*10 and 26 connecting edges. These edges refer to prior-knowledge interactions between connected proteins. First, the network is transformed into an undirected labeled graph *G*(*V*, *E*) (Fig. 3B). The graph *G* consists of 10 vertices *V*(*G*) =  {*v*_1_, …, *v*_10_} and 26 edges *E*(*G*) = {*e*_1_, …, *e*_26_}. Each vertex is labeled with one protein (*p*1–*p*10). Notably, a vertex can be labeled with more than one protein. In such case, the proteins in the label form a clique in the PPIN (e.g., vertex *p*1, *p*2, *p*4 in Fig. 3D). Two vertices *v*_*x*_ and *v*_*y*_ (with *x*, *y* ∈ 1, …, 10 and *x* ≠ *y*) are connected by an edge if there is at least one known relation in the PPIN between the proteins represented by *v*_*x*_ as well as the proteins represented by *v*_*y*_. The weight of the edge connecting *v*_*x*_ and *v*_*y*_ denotes for the number of relations between the proteins represented by *v*_*x*_ and the proteins represented by *v*_*y*_. Initially, all edges have weight 1.

Phase 2 of Step I: Identification of minimal cliques of size three: Starting with randomly selected seed proteins, the algorithm first identifies minimal cliques of size three. A seed is dropped if it is not part of a minimal clique. In Fig. 3(C), we start with *p*4 (colored red) as a seed and search for any minimal clique of size three by exploring its neighbors (colored yellow) as well as their neighbors. The order in which vertices are explored is random. In our example, the first clique identified is formed by *p*1, *p*2 and *p*4 and the corresponding vertices are merged into the vertex *p*1, *p*2, *p*4 (Fig. 3D). Next, the weights of the edges are updated. In our example (Fig. 3D), the edge between *p*1, *p*2, *p*4 and *p*3 is now weighted 3, since the proteins *p*1, *p*2 and *p*4 are all connected to protein *p*3 (Fig. 2A). The edge’s weight connecting *p*1, *p*2, *p*4 with *p*5 remains 1, since only *p*4 is connected to *p*5. Following the same strategy, the minimal clique *p*5, *p*6, *p*7 is identified starting from the seed *p*6 (Fig. 3D) while the seed *p*9 is merged with *p*8 and *p*10 into *p*8, *p*9, *p*10 (Fig. 3E). All edge weights are updated accordingly.

Phase 3 of Step I: Extension of all minimal cliques: All minimal cliques of size three (Fig. 3E; green) are now iteratively extended in random order until they cannot be enlarged further. Once a node becomes part of a clique, it cannot become part of another clique, i.e., cliques compete for nodes in the graph. Starting from Fig. 3(E), *p*1, *p*2, *p*4 is processed first. *p*1, *p*2, *p*4 is connected to *p*3 by an edge of weight 3. Thus, all proteins *p*1, *p*2 and *p*4 are connected to *p*3 (Fig. 3A). Therefore, both vertices can be merged to form the new vertex *p*1, *p*2, *p*3, *p*4 (Fig. 3F). Next, the clique represented by *p*5, *p*6, *p*7 is processed. The edge connecting *p*5, *p*6, *p*7 with *p*8, *p*9, *p*10 has a weight of 9. This indicates that all proteins of *p*5, *p*6, *p*7 are connected with all proteins of *p*8, *p*9, *p*10. Therefore, both vertices are merged to form *p*5, *p*6, *p*7, *p*8, *p*9, *p*10 (Fig. 3F). Finally, no clique can be enlarged any further. The algorithm terminates reporting two cliques, i.e., the clique formed by the proteins *p*1, …, *p*4 as well as the clique formed by the proteins *p*5, …, *p*10.

Phases 1–3 of step I of the algorithm are repeated for *n* iterations with random seed proteins in each iteration until the set of obtained cliques sufficiently approximates the community structure underlying the PPIN.

#### Step II: Identification of significantly enriched cliques

In step II (Fig. 2II) all enumerated cliques are tested for their enrichment with phenotype-associated proteins, e.g., proteins corresponding to DEGs from high-throughput gene expression data (Fig. 2, Input). The p-value for each clique is calculated using a permutation-based test^38^. In detail, for a gene expression platform measuring *N* genes, with *D* ∈ *N* being the set of DEGs, the gene sets *B* are created, each containing |*D*| genes sampled from *N*. For each clique in *C*, the p-value *p*_*i*,*D*_ of clique *c*_*i*_ (*i* = 1, …, |*C*|) is calculated using the one-sided Fisher’s exact test. Accordingly, the p-value *p*_*i*,*b*_ of clique *c*_*i*_ is calculated for each gene set *b* in *B*. The final p-value ${p}_{i}^{\ast }$ is then calculated according to equation 1.

1 $${p}_{i}^{\ast }=\frac{|\forall B:{p}_{i,b}\le {p}_{i,D}|}{|B|}$$

#### Step III: Assembly of the regulatory module

Based on an user-defined p-value cutoff we filter significantly enriched cliques. Since cliques can overlap in their proteins, the union of all significantly enriched cliques (Fig. 2III) results in a large regulatory module (Fig. 2, Output). This module summarizes biological processes and molecular mechanisms underlying the respective phenotype.

#### Reproducibility of regulatory modules

ModuleDiscoverer is a heuristic that approximates the underlying community structure. Since the exact solution is unknown, quality of the approximation cannot be assessed directly. Instead, we can test if additional iterations of the algorithm, i.e., the enumeration of more cliques, has a qualitative impact on the regulatory module in terms of additional nodes and edges. To this end, non-parametric bootstrapping sampling (with replacement) is applied to assess reproducibility of the regulatory module. Based on the results of *n* iterations of ModuleDiscoverer, we create bootstrap samples of *n* iterations and identify the respective regulatory modules. Pairwise comparison of the regulatory modules in terms of shared edges and nodes then provides a distance between the two regulatory modules. The median of all distances divided by the average number of nodes and edges reflects the stability of the regulatory module. See Supplementary File S1 section 1.4 for details.

### ModuleDiscoverer: application to biological data

To demonstrate the application of ModuleDiscoverer we used the PPIN of *R. norvegicus* in conjunction with gene expression data of a rat model of diet-induced NASH for the identification of a NASH-regulatory module. The results will be presented in three sections: (i) processing of the PPIN (Fig. 2, I), (ii) identification of significantly enriched cliques based on high-throughput expression data (Fig. 2, II) and iii), assembly of the regulatory module based on the union of all significantly enriched cliques (Fig. 2, III). Finally, the NASH-regulatory module will be analyzed and validated.

#### Processing of the PPIN

The PPIN of *R. norvegicus* (STRING, version 10) was filtered for high-confidence relations with a score >0.7. This retained 15,436 proteins connected by 474,395 relations. Next, we used the single-seed approach of ModuleDiscoverer to enumerate maximal cliques using 2,000,000 iterations. This identified 1,494,126 maximal cliques in total, enclosing 185,178 unique maximal cliques. Additionally, we applied ModuleDiscoverer with 1,020,000 iterations using the multi-seed approach with 25 seed proteins per iteration. This resulted in 18,807,344 cliques in total enclosing 2,269,022 unique cliques.

#### Identification of significantly enriched cliques

Based on the expression data, we identified 286 DEGs (p-value < 0.05) out of 4,590 EntrezGeneID-annotated genes on the microarray platform (Supplementary File F2). 10,000 data sets were created sampling 286 random genes out of 4,590 genes in the statistical background. Finally, genes of all data sets were translated into EnsemblProteinIDs using the R-package *org.Rn.eg.db*.

P-value calculation according to equation 1 was performed for each clique satisfying the following two properties. First, at least one protein in the clique is associated to a DEG. Second, at least half of the proteins in the clique are associated to genes in the statistical background. For the p-value cutoff 0.01 we identified 696 significantly enriched cliques for the single-seed approach and 5,386 significantly enriched cliques for the multi-seed approach. Notably, permutation-based calculated p-values were similar to p-values calculated using the one-sided Fisher’s exact test (Supplementary Figure F1).

#### Assembly and analysis of the regulatory module

**Figure 4:**
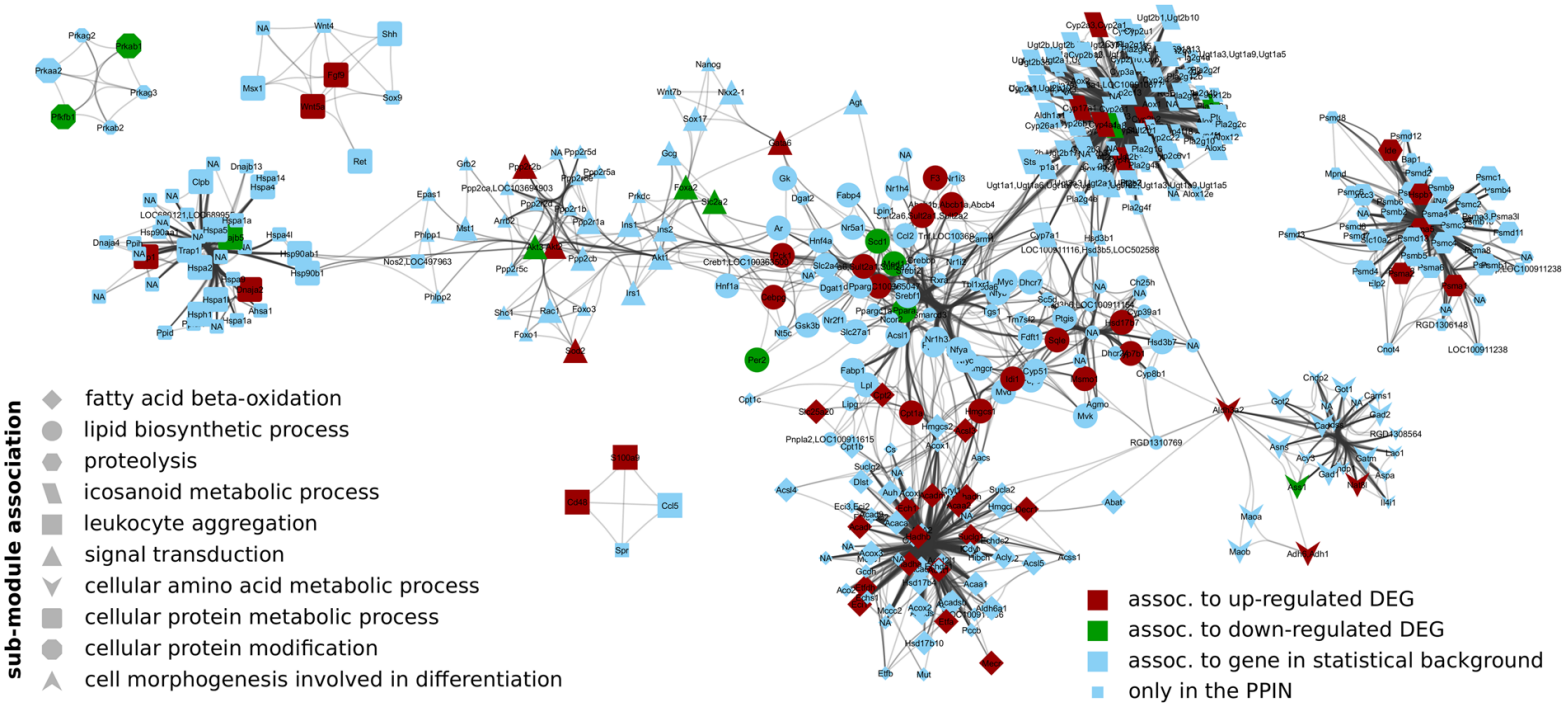
The identified NASH-regulatory module. Nodes (proteins) are labeled with the official gene symbol. Their membership in a sub-module is shape-coded.

The single-seed regulatory modules contains five disconnected sub-networks composed of 311 proteins connected by 3,180 relations. 175 of the 311 proteins are associated to background genes and 60 are associated to DEGs. Similar, the regulatory module of the multi-seed approach contains five sub-networks composed of 415 proteins and 4,975 relations in total (Fig. 4). 210 of these 415 proteins are associated with background genes and 67 proteins are associated with DEGs. Both of the regulatory modules are significantly enriched (*p* < 10^−4^) with proteins associated to DEGs. Based on 100 bootstrap samples we found that both regulatory modules are reproducible with an average variability of less than 5% (Supplementary Figure F2). Furthermore, we investigated the robustness of the modules to changes in the edge score cutoff of the PPIN, i.e., the robustness of the algorithm to noise in the PPIN. We found that both regulatory modules are composed of a reproducible set of core proteins (Supplementary File S1), which contribute to a strong similarity among these regulatory modules compared with the similarity to regulatory modules identified with other algorithms. Apart from a single edge, the multi-seed regulatory module encloses the single-seed regulatory module. Thus, we decided to focus on the multi-seed regulatory module as an extension to the single-seed regulatory module.

Next, we identified pathways significantly enriched with proteins for the regulatory module shown in Fig. 4. The results (Supplementary File S3) highlighted NASH-relevant pathways such as fatty acid degradation and elongation, PPAR signaling pathway^39^, arachidonic acid metabolism^40^, the metabolism of diverse amino acids^41^ as well as insulin signaling pathway^42,43^. Identification of sub-modules based on the edge-betweenness centrality measure^44^ in the network revealed 10 sub-modules. These sub-modules are sparsely connected with each other but densely connected within themselves. In Fig. 4, the sub-module membership of each protein (and thus its associated biological process) is shape-coded. We performed an enrichment analysis for the proteins of each sub-module to identify its potential biological functions (Supplementary File S4).

We found that the most central sub-module (Fig. 4, circles) is associated with the lipid biosynthetic process. For example, the KEGG PPAR-signaling pathway is significantly enriched with proteins from the module. This pathway plays a key-role in the development of FLD by regulating the beta-oxidation of fatty acids, the activation of anti-inflammatory pathways and the interaction with insulin signaling^45^. In agreement with these findings, the sub-module is directly connected to sub-modules associated to fatty acid beta-oxidation (diamonds), icosanoid-metabolic processes (parallelogram) and cellular signal transduction such as the insulin signaling pathway (triangles). Another directly connected sub-module is associated to the metabolism of cellular amino acids (V-shaped) such as alanine, aspartate and glutamate metabolism as well as phenylalanine, tyrosine and tryptophan metabolism.

Another two sub-modules are associated to proteolysis (hexagons) and the metabolism of cellular proteins (round rectangle) with the latter being directly connected to the sub-module associated with signal transduction (triangles). The connection between cellular protein metabolic processes such as the response to unfolded proteins (Supplementary File S4, sub-module 8) and NAFLD as well as NASH has been studied extensively and is reviewed in^46^.

#### Detection of regulatory modules using module cover approaches

We compared the identified NASH-regulatory module with the regulatory modules identified by three ‘module cover algorithms’ (see Batra *et al*.^31^), namely MATISSE, DEGAS and KeyPathwayMiner (see methods for details).

The identified modules were compared based on EnsemblProteinIDs and results are summarized in Table 1. We found that DEGAS produced the smallest module composed of 42 proteins, followed by KeyPathwayMiner with 100 proteins. The modules produced by MATISSE (314) and ModuleDiscoverer (single-seed: 311; multi-seed 415) are similar in size. With app. 24%, the modules of MATISSE and KeyPathwayMiner show the highest overlap with the set of proteins associated to all DEGs, followed by ModuleDiscoverer (app. 9%) and DEGAS (app. 2%). The regulatory module by MATISSE overlaps with the modules of ModuleDiscoverer and KeyPathwayMiner to about 22%–26%. The module of KeyPathwayMiner overlaps with the modules of ModuleDiscoverer by app. 13%–16%. Thus, modules produced by ModuleDiscoverer are more related to the modules produced by MATISSE compared to KeyPathwayMiner.

**Table 1:** Node-wise overlap between identified regulatory modules of DEGAS, MATISSE, KeyPathwayMiner (KPM), ModuleDiscoverer single-seed (MD-SS) and multi-seed (MD-MS) as well as the set of DEG-associated proteins, i.e., differentially regulated proteins (DRPs).

	DRPs	MD-SS	MD-MS	DEGAS	MATISSE	KPM
DRPs	410	9.08%	8.84%	2.26%	23.55%	23.79%
MD-SS		311	74.49%	3.22%	22.79%	15.77%
MD-MS			415	2.47%	21.50%	13.44%
DEGAS				42	3.19%	8.40%
MATISSE					314	26.22%
KPM						100

The overlap (given in %) is defined as fraction of the intersection of the module’s nodes from the union of the module’s nodes. The diagonal of the matrix contains the total number of proteins in the module.

**Figure 5:**
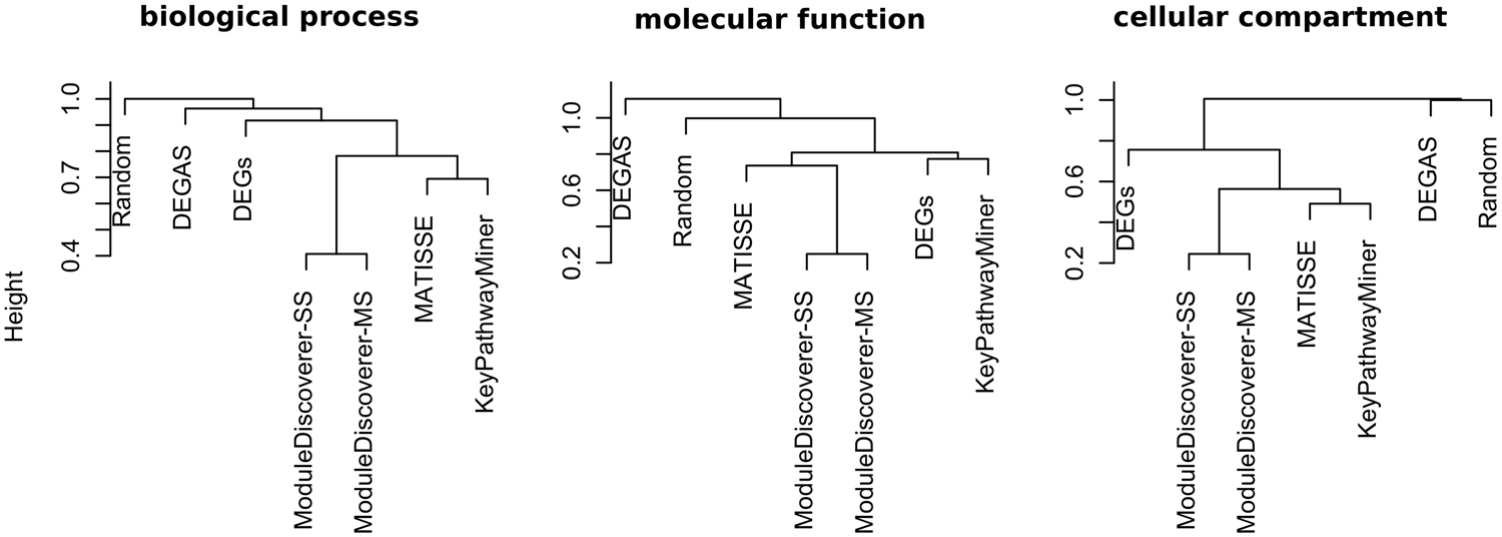
Similarity of modules given by the correlation-based distance measure of ranked lists of significantly enriched GO-terms. The height corresponds to the correlation-based distance (see methods), where values <1 denote for a positive average correlation.

Next, we were interested in the module’s mutual agreement regarding the underlying biology. Hierarchical clustering was used to visualize the correlation-based distance measure (see methods) between regulatory modules obtained from lists of significantly enriched GeneOntology (GO)-terms. Figure 5 outlines the results for the ontologies biological process (BP), molecular function (MF) and cellular compartment (CC). Compared to random lists of GO-terms (Fig. 5, Random), KeyPathwayMiner, MATISSE and ModuleDiscoverer show a positive average correlation for all three ontologies. For BP and CC (Fig. 5, left and right) the regulatory modules of KeyPathwayMiner and MATISSE show a higher agreement in the derived GO-term lists compared to ModuleDiscoverer. With respect to MF (Fig. 5, middle), the GO-term list of the KeyPathwayMiner module shows a high correlation with the GO-term list derived from the set of DEGs. The GO-term list of the MATISSE module are correlated with the GO-term lists of both ModuleDiscoverer modules. Overall, GO-term lists derived from the modules of MATISSE, KeyPathwayMiner as well as ModuleDiscoverer show a positive average correlation with the GO-term lists derived from the set of DEGs.

#### Literature validation of the regulatory module

We corroborated both NASH-modules (single-seed and multi-seed) using curated disease-to-SNP associations (see methods). Disease-to-SNP associations are based on DNA-sequence information. Thus, they can be considered independent from the gene expression data used to identify the module. In contrast to the set of DEGs as well as the set of proteins captured by the modules identified using DEGAS, MATISSE or KeyPathwayMiner, we found that both NASH-modules are significantly enriched (p-value <0.05) with genes associated to NAFLD-relevant SNPs (Supplementary File S5).

**Figure 6:**
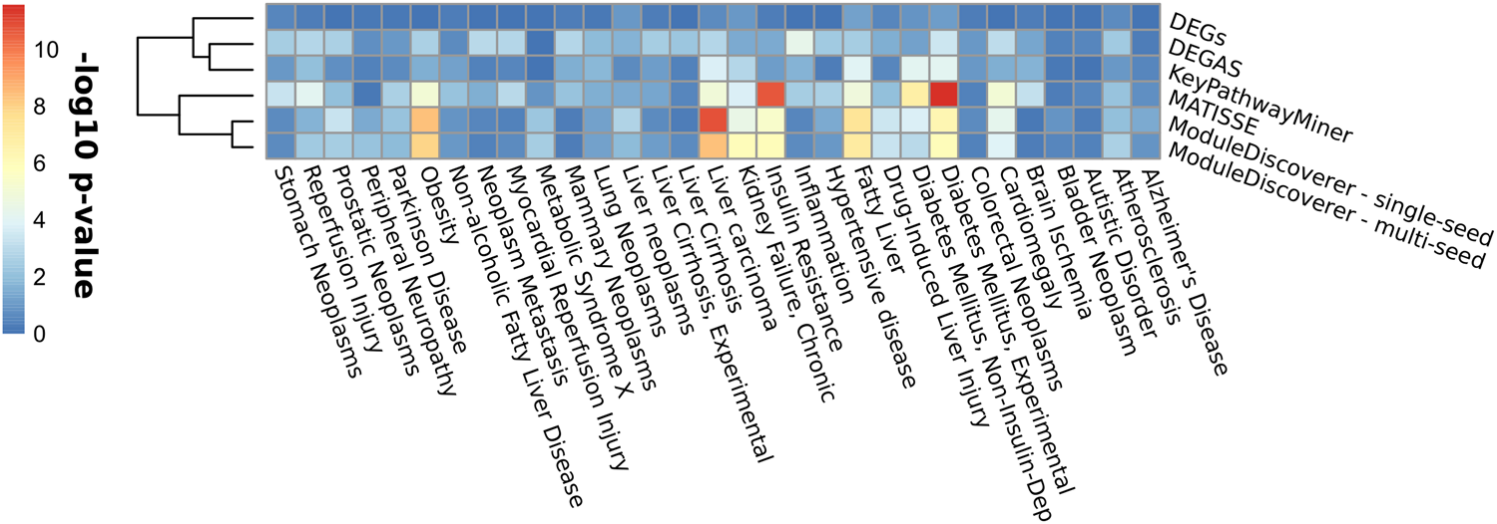
Enrichment of FLD-related diseases with proteins of modules produced by ModuleDiscoverer (single-seed and multi-seed), DEGAS, KeyPathwayMiner and MATISSE as well as the set of DEGs. Higher values equal lower p-values.

Next, we performed a gene enrichment analysis using a list of curated disease-to-gene associations (see methods). The results are outlined in Fig. 6. Both of our NASH-modules show significantly enriched FLD-associated diseases such as obesity, (non-insulin dependent) diabetes mellitus type-2, liver carcinoma and insulin resistance. Notably, for the set of DEGs almost all of these disease-terms (with the exception of ‘Fatty liver’) show a slight, but non-significant enrichment (p-value ≥ 0.05). Compared to ModuleDiscoverer, the modules produced by KeyPathwayMiner and MATISSE show increasing similarity to the results of ModuleDiscoverer.

## Discussion

We have presented ModuleDiscoverer, an algorithm for the identification of regulatory modules based on large-scale, whole-genome PPINs and high-throughput gene expression data. To show applicability of the algorithm, we identified a non-alcoholic steatohepatitis (NASH)-regulatory module for which we relied on the STRING resource only. STRING integrates information from a variety of resources, such as primary interaction databases, algorithms for interaction prediction, pathway databases, text-mining and knowledge transfer based on orthology. Reported relations are thus based on known physical interaction as well as associative information. To ensure quality of the relations, we selected a high cutoff (>0.7) for the combined edge score. Additionally, we found that a small increase/decrease of the selected cutoff has no substantial effect on our results. To further assure robustness of the identified regulatory modules, a comparison of the modules based on different PPINs should be considered. If working with human data, for example, our algorithm could be applied to the human signaling network provided by the Wang Lab^47^. If there is no comparative PPIN or even no PPIN at all for the organism of interest, a yet to explore alternative might be the use of whole-genome gene regulatory networks (GRNs). Algorithms such as presented in Altwasser *et al*.^48^ are based on mathematical models that combine expression data and prior-knowledge interaction data. In such GRNs, relations denote for functional relationships between genes/proteins acting in common biological contexts, which equals networks derived from STRING^37^. This corresponds to the idea of regulatory modules as shown in Fig. 1.

We compared the ModuleDiscoverer-identified NASH-modules to the modules detected by DEGAS, KeyPathwayMiner and MATISSE. Based on the comparison of rank-transformed lists of significantly enriched GO-terms, the DEGAS-, KeyPathwayMiner-, MATISSE- and ModuleDiscoverer-produced modules as well as the set of DEGs correlate in their underlying biology. Interestingly, the module by MATISSE (followed by KeyPathwayMiner) overlaps most with the ModuleDiscoverer-identified module. This can be explained by the methodology underlying the algorithms. KeyPathwayMiner identifies connected sub-networks of proteins associated to DEGs. Exception nodes, i.e., nodes not associated to DEGs, are included as ‘bridges’ to identify the overall maximal connected sub-network. Thus, modules by KeyPathwayMiner are always centered around proteins associated to DEGs. In contrast, MATISSE calculates weights for the PPIN’s edges based on a probabilistic model estimating the similarity between proteins given the underlying expression data. Proteins without expression information do not contribute to the score during the module finding process. Thus, MATISSE-identified modules contain also peripheral exception nodes. This relates to the ‘guild-by-association’ principle of ModuleDiscoverer, which includes an exception gene in the module if a significant amount of measured genes in its direct neighborhood, i.e., the set of genes that form the maximal clique, is associated to a DEG. In contrast to MATISSE however, the clique assumption by ModuleDiscoverer naturally limits the number of exception nodes to those that are part of the clique. In consequence, we cannot state the best performing algorithm since the results strongly depend on the underlying assumptions. However, based on the validation, we found that only the ModuleDiscoverer-identified NASH-modules contain a significant number of proteins associated to NAFLD-relevant SNPs.

We find that the identified NASH-module (Fig. 4) reflects the experimental clinical and histological observations by Baumgardner *et al*. For example, the NASH-module highlights the disease-term ‘Obesity’ as significantly enriched with proteins of the module (Fig. 5). In agreement, Baumgardner *et al*.^36^ observed a significant increase in body weight in the treatment group compared to control (p ≤ 0.05). Moreover, they reported a significant increase in fat mass as percentage of body weight between treatment and control reflecting adiposity. Additionally, serum leptin levels were observed to be significantly increased in the treatment group. The serum leptin level is a marker that positively correlates with obesity^49^. Other significantly enriched disease terms include ‘Insulin Resistance’, ‘Diabetes Mellitus Type-2’ and ‘Diabetes Mellitus, Experimental’. Baumgardner *et al*.^36^ reported significantly increased serum insulin concentrations compared to control rats that were overfed with a high-fat 5% corn oil diet at (220 kcal*kg ^−3/4^*day ^−1^~17%) for 21 days. They concluded that this observation points towards hyperinsulinemia, which can be due to insulin resistance and is often associated with type-2 diabetes. Finally, we found the disease-term ‘Fatty Liver’ significantly enriched in proteins of the module. Baumgardner *et al*.^36^ reported that histological examination of the liver samples showed steatosis, macrophage infiltration and focal necrosis in the treatment samples. This was accompanied by significantly elevated serum alanine aminotransferase (ALT) levels and significantly increased serum and liver triglyceride concentrations. Notably though, other inflammation-associated scores such as hepatocellular ballooning and lobular inflammation/necrosis were reported to be elevated but not statistically significant. This could explain the non-significantly enriched disease-terms such as ‘Inflammation’ and ‘Liver Cirrhosis’.

To further evaluate our algorithm, we used a small sub-network of the high-confidence PPIN of *R. norvegicus* (Supplementary File S1). We showed that the single-seed approach as well as the multi-seed approach work well in principle and highlighted their advantages as well as disadvantages. In summary, in cases where large-scale, genome-wide PPINs cannot be processed by MCE-solving algorithms, i.e., the regulatory module based on the exact solution cannot be determined, the use of ModuleDiscoverer becomes inevitable. In such situations, the regulatory module of the single-seed and the multi-seed approach should be identified. While single-seed-based regulatory module is more consistent with results of MCE-based approaches, the multi-seed regulatory module will extend the single-seed based regulatory module with proteins that may have been missed due to a PPIN structure of highly overlapping maximal cliques.

## Conclusion

We presented ModuleDiscoverer, a heuristic approach for the identification of regulatory modules in large-scale, whole-genome PPINs. The application of ModuleDiscoverer becomes favorable with increasing size and density of PPINs. Compared to the MCE-based approach, we demonstrated that ModuleDiscoverer identifies modules that can be identical (single-seed approach) or even more comprehensive (multi-seed approach). We applied our algorithm to experimental data for the identification of the regulatory module underlying a rat model of diet-induced NASH. The identified NASH-regulatory module is stable, biologically relevant, reflects experimental observations on the clinical and histological level and is comparable to the results of three published module detection algorithms. In contrast to any of the modules identified by these algorithms or the set of DEGs alone, our NASH-module is significantly enriched with NAFLD-associated SNPs derived from independent GWASs. Altogether, we consider ModuleDiscoverer a valuable tool in the identification of regulatory modules based on large-scale, whole-genome PPINs and high-throughput gene expression data.

## Methods

### Microarray data, pre-processing and differential gene expression analysis

Affymetrix microarray gene expression data of a rodent model of diet-induced NASH published by Baumgardner *et al*.^36^ was downloaded from Gene Omnibus Express^50^ (GSE8253). In brief, the animal model was obtained by overfeeding rodents with a high-fat diet based on 70% corn oil at moderate caloric excess (220 kcal*kg ^−3/4^*day ^−1^~17%) for 21 days via total enteral nutrition (TEN)^36^. They compared the treatment group against a control group of rats fed a diet based on 5% corn oil at normal caloric levels (187 kcal*kg ^−3/4^*day ^−1^) for 21 days via TEN. Gene expression in each experimental group was measured using three microarrays.

Affymetrix Rat Genome U34 arrays were annotated with custom chip definition files from Brainarray version 15^51^. Raw data was pre-processed using RMA^52^. Differential gene expression was assessed using *limma* ^53^ with a p-value <0.05 (Supplementary File S2).

### SNP-gene-disease and gene-disease association data

Disease-to-SNP relations as well as curated disease-to-gene associations for *H. sapiens* were obtained from DisGeNET^54^. All text-mining based disease-to-SNP associations were removed. Furthermore, we removed all associations involving genes without an orthologue in *R. norvegicus*. Orthology information was obtained from the RGD^55^. For the disease-to-gene associations we created a disease network similar to Goh *et al*.^56^. In this network, two diseases (nodes) are connected if they share ≥10 genes. Selecting the first neighbors of the terms ‘Fatty Liver’ and ‘Non-alcoholic Fatty Liver Disease’ yielded a list of 31 NAFLD-relevant diseases.

### Algorithms for phenotype-specific module identification

We tested three different phenotype-specific module identification algorithms named MATISSE^32^, DEGAS^33^ and KeyPathwayMiner^34^. MATISSE and DEGAS are implemented in the MATISSE toolbox^57^. For KeyPathwayMiner we downloaded the stand-alone application (version 4.0)^58^. For all algorithms, the high-confidence interactome of *R. norvegicus* from STRING was converted to *sif*-format. EntrezGeneID-based gene identifiers of the microarray were converted to EnsemblProteinIDs using the *org.Rn.eg.db* database.

#### Matisse

Matisse aims at the identification of connected components (connected sub-networks) composed of nodes associated with genes of high similarity, e.g., genes with similar expression profiles. MATISSE starts from small, high-scoring groups of proteins (as defined by a probabilistic model estimating the similarity between genes). These seed groups are step-wise modified (extended, reduced, exchanged or merged) until the overall score is maximized. We applied MATISSE to expression data of all six samples (three control, three case) for all DEGs. Starting from seed protein groups with minimal/maximal size of 5/50, MATISSE was run to identify regulatory modules with minimal/maximal size of 5/100. Pearson correlation was used to assess similarity between gene expression patterns (default parameter settings). A total of four regulatory modules was identified, which we combined into a single regulatory module for further analysis.

#### Degas

Degas aims at the identification of minimal (*k*, *l*)-components (connected sub-networks) where at least *k* genes are differentially expressed in all but *l* cases. The algorithm was run using expression data of all six samples (three control, three case) for the full set of genes available on the microarray. The CUPS heuristic was used to identify all regulatory modules with at least *k* = 40 genes differentially expressed (p-value <0.05) in all but *l* = 1 case. *k* was optimized automatically within a range of 10 and 50 using *k*-steps of 10 (default parameter settings). The algorithm identified one regulatory module, which was used for further analysis.

#### KeyPathwayMiner

KeyPathwayMiner identifies maximal (*k*, *l*)-components (connected sub-networks) with at most *k* genes that are not differentially expressed in all but *l* cases. The algorithm was applied using the full set of genes available in the data. Instead of using expression data for all six samples we provided an indicator flag (0/1) to mark differentially expressed genes (1). The algorithm identified regulatory modules containing a maximum of *k* = 2 genes, which are not differentially expressed (*l* = 0) using the INES strategy. The best-scoring module was selected for further analysis.

### Comparing modules based on lists of GO-term

The distance between regulatory modules from different algorithms was estimated based on the correlation of ranked lists of significantly enriched GO-terms. For each identified regulatory module we performed a gene enrichment analysis using GOstats with the *org.Rn.eg.db* package. P-values > = 0.05 were set to 1 and p-value-ordered GO-term lists were rank-transformed. Indices corresponding to ties were ordered at random. The ranking was repeated 1,000 times. Spearman’s rank correlation coefficient was calculated for each repeat. The final correlation between the GO-term lists of two methods was averaged over all 1,000 repeats. We defined the distance as 1 minus the correlation coefficient.

## Electronic supplementary material

Supplementary information

Supplementary File S2

Supplementary File S3

Supplementary File S4

Supplementary File S5
