# Supplementary material for: ModuleDiscoverer: Identification of regulatory modules in protein-protein interaction networks
Source: Sci Rep. 2018 Jan 11;8:433. doi: 10.1038/s41598-017-18370-2 (PMC5764996; doi:10.1038/s41598-017-18370-2)
Supplement: Supplementary file 1 — Supplementary information [file 41598_2017_18370_MOESM1_ESM.pdf]

ModuleDiscoverer: Identification of regulatory modules in protein-protein interaction networks.

- supplementary information -

Sebastian Vlaic[1,2,*], Theresia Conrad[1], Christian Tokarski-Schnelle[1,3], Mika Gustafsson[4], Uta Dahmen[3], Reinhard Guthke[1], Stefan Schuster[2]

[1]Leibniz Institute for Natural Product Research and Infection Biology - Hans-Knöll-Institute, Systems Biology and Bioinformatics, D-07745 Jena

[2]Friedrich-Schiller-University, Department of Bioinformatics, D-07743 Jena

[3]University Hospital Jena, Friedrich-Schiller-University, General, Visceral and Vascular Surgery, D-07749 Jena

[4]Bioinformatics, Department of Physics, Chemistry and Biology, Linköping University, SE-581 83 Linköping

*Corresponding author

Contents

1 Performance of ModuleDiscoverer **2**

1.1 The *PPAR-alpha* -centered PPI sub-network of *R. norvegicus* 4
1.2 Computing the reference regulatory module 5
1.3 Motivation for ModuleDiscoverer 7
1.4 Assessing quality of identify regulatory modules 7
1.5 ModuleDiscoverer using the single-seed approach 9
1.6 ModuleDiscoverer using the multi-seed approach 12
1.7 Discussion 17
1.8 Conclusion 19

2 Robustness of ModuleDiscoverer to noise in the PPIN **19**

3 Supplementary Figures **25**

3.1 Supplementary Figure F1 25
3.2 Supplementary Figure F2 26

1 Performance of ModuleDiscoverer

ModuleDiscoverer is an algorithm for the identification of regulatory modules based on protein-protein interaction networks (PPINs) in conjunction with gene expression data from high-throughput experimental data. As opposed to comparable approaches such as presented by Barrenas *et al.* [1], ModuleDiscoverer does not rely on the computationally demanding enumeration of all maximal cliques (the maximal clique enumeration (MCE) -problem) in the PPIN, which is an NP-hard problem [2]. Instead, the presented approach approximates the community structure underlying the network by iterative enumeration of cliques starting from random seed nodes. The number of seed nodes used for the identification of cliques in each iteration has to be chosen manually. In general, there are two specific modes of operation that can be distinguished. Firstly, application of ModuleDiscoverer using exactly one seed node and secondly, the use of two or more seeds. In the following we will describe the algorithm for both modes of operation. The workflow for the evaluation of ModuleDiscoverer is outlined in figure 1.

Based on a small sub-network of the high-confidence PPIN (edge threshold > 0.7) of *R. norvegicus* as obtained from the STRING database [4] and gene expression data by Baumgardener *et. al* [3] (see material and methods in the manuscript), we will first identify a reference regulatory module. Section 1.3 will be used to outline the motivation for the development of ModuleDiscoverer. In section 1.4 we will introduce measures for the comparison of regulatory modules as well as the quantification of their stability. In section 1.5 we will use ModuleDiscoverer to identify the single-seed-based regulatory module and assess its quality based on the measures introduced in section 1.4. Section 1.6 will be used to compare the quality of two regulatory modules using the multi-seed approach with two different numbers of seeds. Finally, in section 1.7 we will discuss our findings.

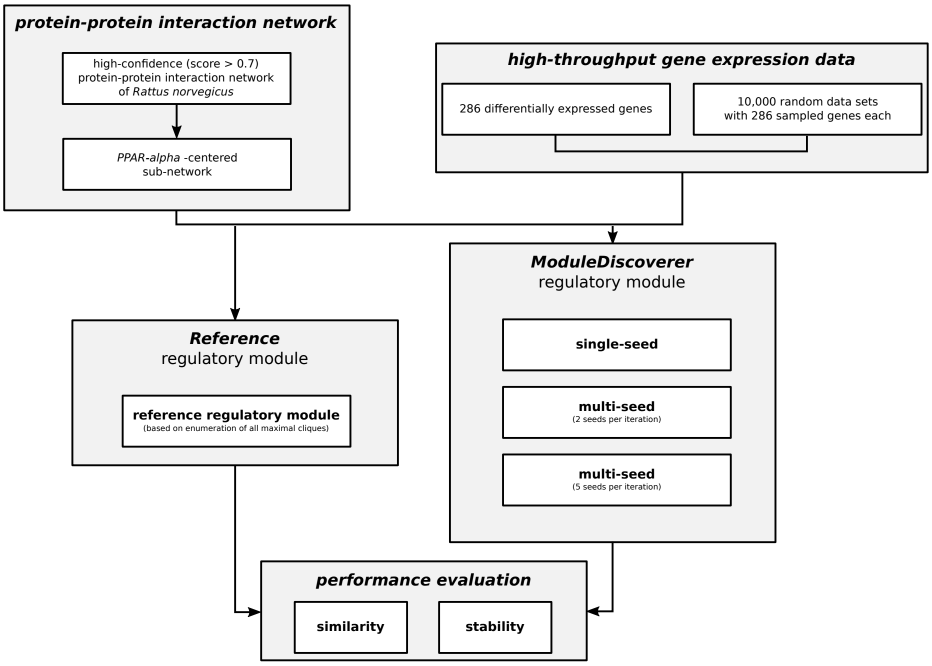

Figure 1: **Workflow for the performance evaluation of ModuleDiscoverer.** Based on the high-confidence *PPAR-alpha* -centered sub-network of *Rattus norvegicus* and gene expression data by [3], a reference regulatory module will be identified. ModuleDiscoverer will then be used to identify the regulatory module based on different numbers of seed proteins. The performance is then evaluated based on the stability of the identified regulatory modules as well as their similarity with the reference regulatory module.

1.1 The *PPAR-alpha* -centered PPI sub-network of *R. norvegicus*

To obtain a sub-network that can be rapidly processed and analyzed, we decided to use the protein *peroxisome proliferator activated receptor alpha* (*PPAR-alpha*) and all of its 277 first neighbors. Altogether, the *PPAR-alpha* -centered sub-network is composed of 4,902 edges connecting 278 nodes. The network, as obtained from STRING, is shown in figure 2.

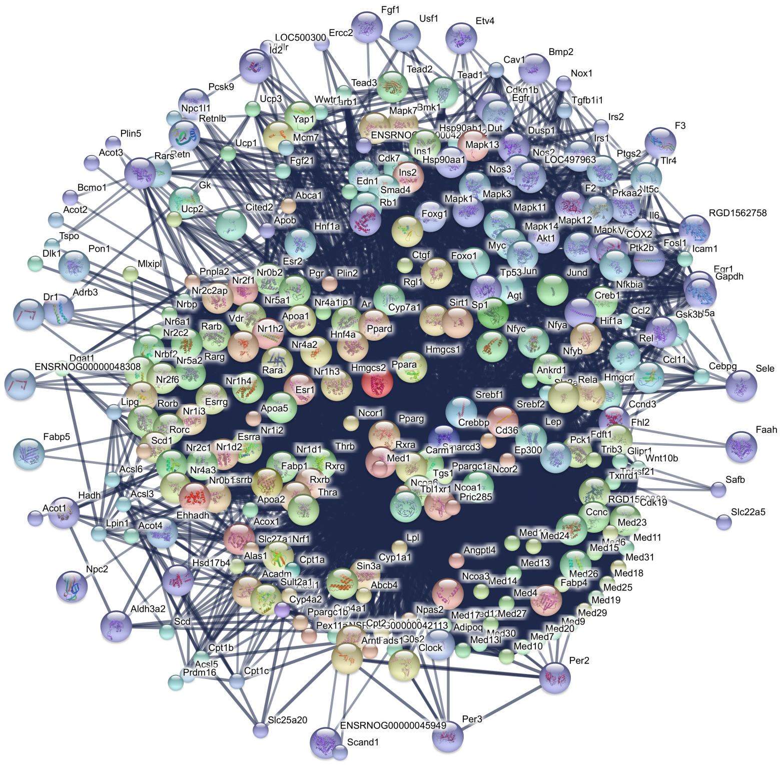

Figure 2: ***PPAR-alpha* -centered PPIN of *R. norvegicus*.** The network is composed of 278 nodes connected by 4,902 high-confidence edges with a STRING-score > 0.7. If available, nodes are labeled with the official gene symbol, else with the corresponding EnsemblGeneId.

We selected *PPAR-alpha* as the center of the sub-network since this transcription factor is a major regulator in fatty liver disease (FLD) [5]. Thus, we can make use of the expression data of the rat model of diet-induced non-alcoholic steatohepatitis (NASH) from Baumgardener *et al.* [3] to test the performance of ModuleDiscoverer. Furthermore, this allows to relate

our findings directly to the results of the manuscript, in which we use this data set for the identification of a NASH-regulatory module using the full high-confidence PPIN of *R. norvegicus*.

1.2 Computing the reference regulatory module

The small size of the *PPAR-alpha* -centered sub-network allows the enumeration of all maximal cliques and thus, to compute the regulatory module similar to the approach presented by Barrenäs *et al.* [1]. In contrast to Barrenäs *et al.*, we omit direct interactions between two proteins, i.e., cliques of size two. Furthermore, instead of p-value calculation using Fisher's exact test, we perform permutation-based p-value calculation as outlined in equation 1 in the manuscript. The identified regulatory module then serves as reference to evaluate the performance of ModuleDiscoverer.
Using the *igraph* package [6] for R [7] we identified 1,341 maximal cliques with size >= three. As outlined in figure 3, most of the enumerated maximal cliques have less then 20 members. The largest maximal clique is of size 45.

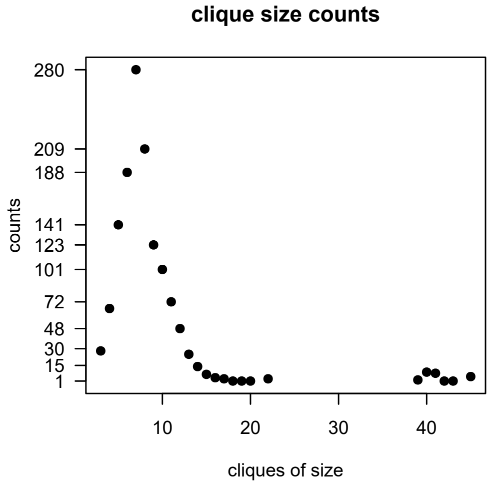

Figure 3: **Number of maximal cliques with size 3 to 45 for the *PPAR-alpha* -centered sub-network.**

Of all 1,341 maximal cliques, we tested 1,057 for their enrichment with proteins associated to the 286 DEGs (Supplementary File S2) identified in the expression data. These 1,057 cliques comprise all maximal cliques with

(i) at least one protein being associated to a DEG and at least half of the proteins being associated to a gene in the statistical background. As a result, we identified 36 cliques significantly enriched (p-value < 0.01). The union of these 36 cliques forms the reference regulatory module shown in figure 4. This network is composed of 60 nodes connected by 282 edges. 42 of these 60 proteins are associated to genes in the statistical background (brown and green nodes). 19 of these proteins are associated to DEGs (green nodes). Based on 10,000 data sets with 286 randomly sampled genes from the statistical background, we find that the regulatory module is significantly enriched with proteins associated to DEGs (p-value $< 10^{-4}$).

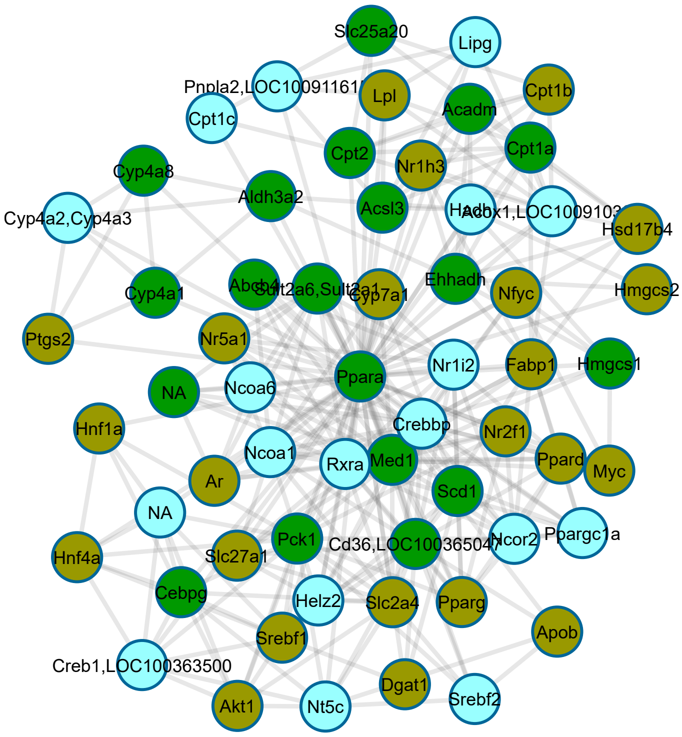

Figure 4: **The reference regulatory module identified based on all 1341 maximal cliques of the *PPAR-alpha* -centered sub-network.** The color of the nodes corresponds to proteins associated with a DEG (green), proteins associated with a gene that was measured in the experiment but is not differentially expressed (brown) and proteins that are within the PPIN but not present on the microarray. Nodes are labeled with the official gene symbol or 'NA', if the official gene symbol is not available.

1.3 Motivation for ModuleDiscoverer

When computing the reference regulatory module we found that only 1,057 of all 1,341 maximal cliques are potentially relevant for the regulatory module. Of these 1,057 only 36 maximal cliques were identified as significantly enriched with proteins associated to DEGs. This shows that depending on the expression data used we are interested in only a subset of all maximal cliques within the PPIN. Consequently, it is straight forward to presume that an approximation of the community structure underlying the PPIN by a randomization-based enumeration of cliques can be sufficient for the approximate identification of the regulatory module. This approach becomes even more attractive if we consider that the enumeration of all maximal cliques is NP-hard and thus, can be infeasable for large-scale, whole-genome PPINs.

1.4 Assessing quality of identify regulatory modules

ModuleDiscoverer is a heuristic approach that is based on the randomized enumeration of cliques from seed nodes. Thus, to gain confidence in the identified regulatory modules, we can assess their quality based on two different approaches. Firstly, if the reference regulatory module is known it can be directly compared against the identified regulatory module. To gain confidence in the calculated similarity, a non-parametric bootstrap approach can be applied comparing the bootstrap-based regulatory modules against the reference regulatory module. Secondly, if the reference regulatory module is unknown a non-parametric bootstrap approach can be applied to estimate the stability of the identified regulatory module.

Similarity to a reference regulatory module

To compare the identified regulatory module G_{md} against a reference regulatory module G_{ref} the graph edit distances $d_{nodes}(G_{md}, G_{ref})$ (equation 1) and $d_{edges}(G_{md}, G_{ref})$ (equation 2) are used. These distances represent the number of node and edge insertions required to transform the identified regulatory module G_{md} such that it fully contains the reference regulatory module G_{ref}. Thus, additional nodes and edges that are present in G_{md} compared to G_{ref} are not penalized. The functions $V(G)$ and $E(G)$ return the full set of nodes/edges in G, respectively.

$$d_{nodes}(G_{md}, G_{ref}) = \mid V(G_{ref}) \setminus V(G_{md}) \mid \tag{1}$$

$$d_{edges}(G_{md}, G_{ref}) = \mid E(G_{ref}) \setminus E(G_{md}) \mid \tag{2}$$

Similarity of the regulatory modules is reported for nodes (equation 3) and edges (equation 4) separately relative to the total number of nodes/edges in the reference regulatory module.

$$similarity_{nodes}(G_{md}, G_{ref}) = 1 - \frac{d_{nodes}(G_{md}, G_{ref})}{\mid V(G_{ref}) \mid} \tag{3}$$

$$similarity_{edges}(G_{md}, G_{ref}) = 1 - \frac{d_{edges}(G_{md}, G_{ref})}{\mid E(G_{ref}) \mid} \tag{4}$$

Stability of an identified regulatory module

In most cases, the reference regulatory module will be unknown. Therefore, quality of the identified regulatory module can not be assessed directly. Instead, a non-parametric (model free re-sampling with replacement) bootstrap approach can be used to estimate the stability of a regulatory module. In other words, we estimated the divergence between the regulatory module identified based on n iterations of ModuleDiscoverer and a hypothetical regulatory module identified by performing additional independent n iterations of ModuleDiscoverer. This is based on the presumption that with increasing number of iterations the identification of cliques important for the regulatory module converges. Consequently, the average distance between all sampling-based regulatory modules G^* can be estimated according to equations for nodes (equation 5) and edges (equation 6) separately. In contrast to the equations 5 and 6, the distance $d^*(G_{md}, G_{ref})$ denotes for node insertions/deletions and edge insertions/deletions that are required to transform one graph into the other.

$$d^*_{nodes}(G_{md}, G_{ref}) = \mid (V(G_{md}) \cup V(G_{ref})) \setminus (V(G_{md}) \cap V(G_{ref})) \mid \tag{5}$$

$$d^*_{edges}(G_{md}, G_{ref}) = \mid (E(G_{md}) \cup E(G_{ref})) \setminus (E(G_{md}) \cap E(G_{ref})) \mid \tag{6}$$

The stability of the regulatory module G identified based on n iterations is then calculated using the sampling based regulatory modules G_i^* and G_j^* with $1 \leq i, j \leq b$ for all b samples according to equation 7 for the nodes and 8 for the edges.

$$stability_{nodes}(G) = 1 - \frac{\underset{1 \leq i,j \leq b;\quad i<j}{\text{median}} d^*_{nodes}(G_i^*, G_j^*)}{\underset{1 \leq i \leq b}{\text{median}} \mid V(G_i^*) \mid} \tag{7}$$

$$stability_{edges}(G) = 1 - \frac{\underset{1\leq i,j\leq b;\quad i<j}{\text{median}} d^*_{edges}(G^*_i, G^*_j)}{\underset{1\leq i\leq b}{\text{median}} \mid E(G^*_i) \mid} \tag{8}$$

Notably, the stability of any identified regulatory module will converge against 1 with an increasing number of iterations. To assess confidence in the estimated stability, we can moreover calculate the upper 95% bound ($CB_{0.95}$) from the pairwise node/edge distances divided by the median number of nodes/edges (the variation) across all sample-based regulatory modules G^*. If $CB_{0.95}(G) = 0$ (which equals a stability value of 1), there remains a 5% chance that the identified regulatory module differs from any regulatory module identified based on n iterations of ModuleDiscoverer.

1.5 ModuleDiscoverer using the single-seed approach

When using ModuleDiscoverer with exactly one seed node, the algorithm will always enumerate either one or no single maximal clique per iteration. No maximal clique is returned only if the seed node is not member of any clique of size three. Consequently, if the number of iterations is sufficiently high, all maximal cliques in the PPIN will be enumerated.

Results of the single-seed approach

Since the number of iterations required for the identification of all maximal cliques is unknown we initially perform 1,000,000 iterations. As a result a total of 971,455 maximal cliques were identified containing all 1,341 maximal cliques in the PPIN. This shows that ModuleDiscoverer is able to identify all maximal cliques in a graph given a large enough number of iterations.
Since the full set of maximal cliques was identified, the resulting regulatory module is identical to the reference module shown in figure 4.

Performance of the single-seed approach

To assess performance of the single-seed approach, we were first interested in the number of iterations needed to identify the reference regulatory module. To this end, we compared the identified regulatory modules based on 14 increasing numbers of iterations against the reference regulatory module. Based on 100 bootstrap samples for each number of iterations (Figure 5) we found that on average the single-seed approach will recover the reference regulatory module, i.e., all nodes and edges of the reference module, in 40,000

iterations. For a 95% confidence at least 150,000 iterations are needed to recall the reference regulatory module.

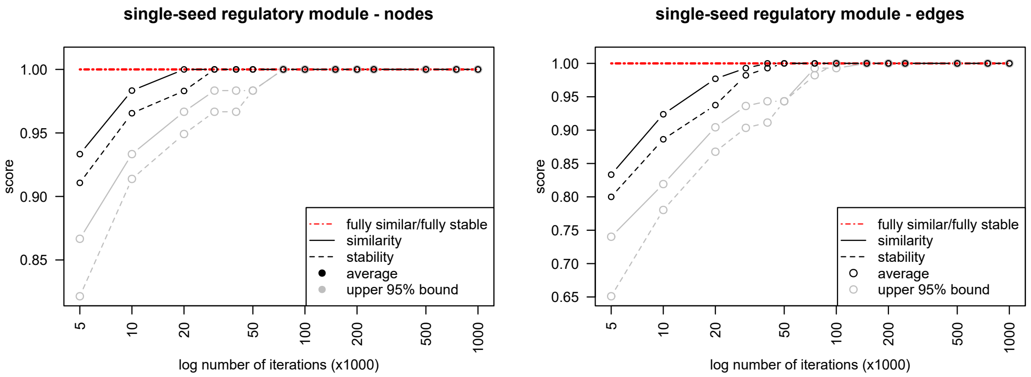

Figure 5: **Performance of the single-seed approach for an increasing number of iterations.** The average (black circles) and upper 95% bound (gray circles) stability of the regulatory module (dashed line) as well as similarity to the reference regulatory module (solid line) are estimated for different numbers of iterations.

Furthermore, based on 100 bootstrap samples we find that the estimated stability of the regulatory module rapidly converges (Figure 5). On average, full stability is reached at 50,000 iterations. However, given the upper 95% bound app. 150,000 iterations are needed to reach stability.
A possible explanation for these large numbers is the high-overlap of maximal cliques in the PPIN. In such networks, the random selection of seed nodes as well as the random extension of cliques favors the identification of large cliques. For the *PPAR-alpha* -centered sub-network this effect can be visualized by plotting the size of each of the 1,341 maximal cliques against the number of occurrences (Figure 6). We find that there are five cliques of size 45 among the 1,341 maximal cliques. Together, these five cliques were identified in app. 19 percent of 1,000,000 iterations. This leads to the identification of mainly the same proteins as well as relations between them. Thus, in dense regions of highly overlapping cliques, proteins of small cliques are less likely to be discovered. Consequently, they are less likely to contribute to the identified regulatory module.

To further illustrate this effect, we use an additional minimal example of a PPIN composed of 12 proteins and 51 edges (Figure 7). In this network,

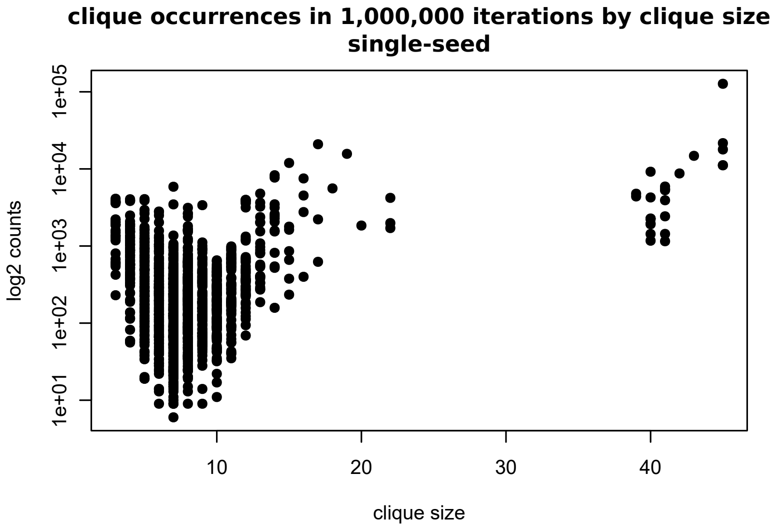

Figure 6: **Clique occurrence by size.** For each of the 1341 maximal cliques (dots), the plot shows its size against the number of occurrences in 1,000,000 iterations.

the 12 proteins are grouped in three overlapping cliques of size 10 (purple), five (blue) and three (green).

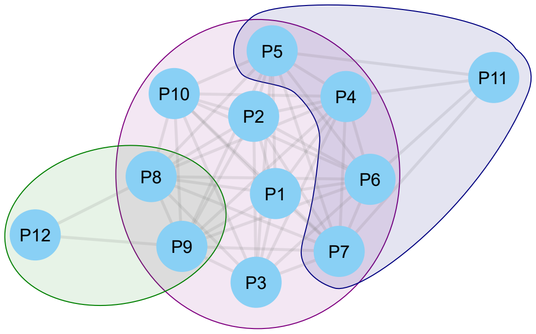

Figure 7: **Maximal cliques in a minimal PPI network.** Proteins in the network ($P1, \ldots, P12$) are grouped in three maximal cliques of size 10 (purple), five (blue) and three (green) respectively.

Based on 10,000 iterations of ModuleDiscoverer we found that the single-seed approach favors the identification of large cliques compared to smaller

cliques. The clique of size 10 was identified in app. 77% of all iterations, while the two smaller cliques were identified at app. 13% (size five) and app. 10% (size three) respectively. Consequently, the proteins $P11$ (Figure 7; blue clique) and $P12$ (Figure 7; green clique) were identified less frequent ($P11$: 1.54%; $P12$: 1.12%) compared to the proteins $P1$ to $P10$ (~9.73%).

1.6 ModuleDiscoverer using the multi-seed approach

The multi-seed approach is intended to avoid the identification of mainly large cliques, a problem we described for the single-seed approach. The use of two or more seed nodes in the PPIN for sequential extension of multiple cliques leads to a shift towards the identification of smaller cliques and consequently, a higher probability for the discovery of the respective proteins. Since cliques compete for nodes in the PPIN (see main manuscript for a detailed description of the algorithm), the algorithm will report maximized cliques without them being necessarily maximal.

Concept of the multi-seed approach

We will illustrate the idea of the multi-seed approach using the minimal PPIN shown in figure 7. Figure 8 provides the result of two arbitrary iterations n and m using ModuleDiscoverer with three seed-nodes per iteration. For both solutions, membership of the proteins to a certain clique is color coded. In iteration m the protein $P12$ is not part of any of the two cliques reported. In this PPIN, the proteins $P1, P2, P3$ and $P10$ will always form a minimal clique, i.e., if one of these proteins is part of an enumerated clique, then all of the remaining three proteins have to be part of that clique, too. Similar, the remaining two maximal cliques (Figure 7, blue and green) have to include at least the proteins $P12, P8$ and $P9$ (green clique), and $P11$ (blue clique). Since a clique is composed of a minimum of three proteins, $P11$ has to group with at least two of the four proteins $P4, P5, P6$ and $P7$. Altogether, this sums up to a total of 25 possible cliques when using two seeds and 36 possible cliques when using three or more seeds per iteration (Figure 9). Of these 25 (36) cliques, 13 (24) cliques contain $P1, P2, P3$ and $P10$, 11 (11) cliques contain $P11$ and one (1) clique is formed by $P8, P9$ and $P12$.

Since the seeds compete for additional nodes during the formation of minimal cliques of size three and their extension, the probability for the identification of one of the cliques containing $P11$ or the clique formed by $P8$, $P9$ and $P12$ increases. Table 1 shows the probability for the identification of one of the 25 (36) possible cliques containing either $P11$ or $P8, P9, P12$

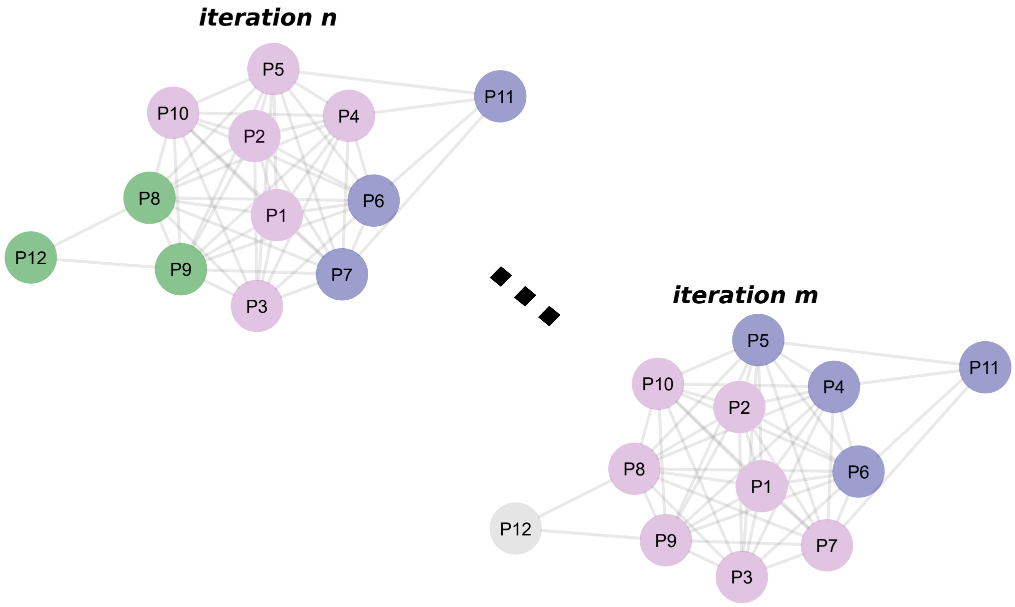

Figure 8: **Two possible solutions for the multi-seed approach using three seed proteins per iteration.** Color (purple, blue and green) of the nodes (proteins) denotes for their membership in a maximized clique. This clique does not have to be necessarily maximal. Gray proteins are not part of any clique reported in the respective iteration.

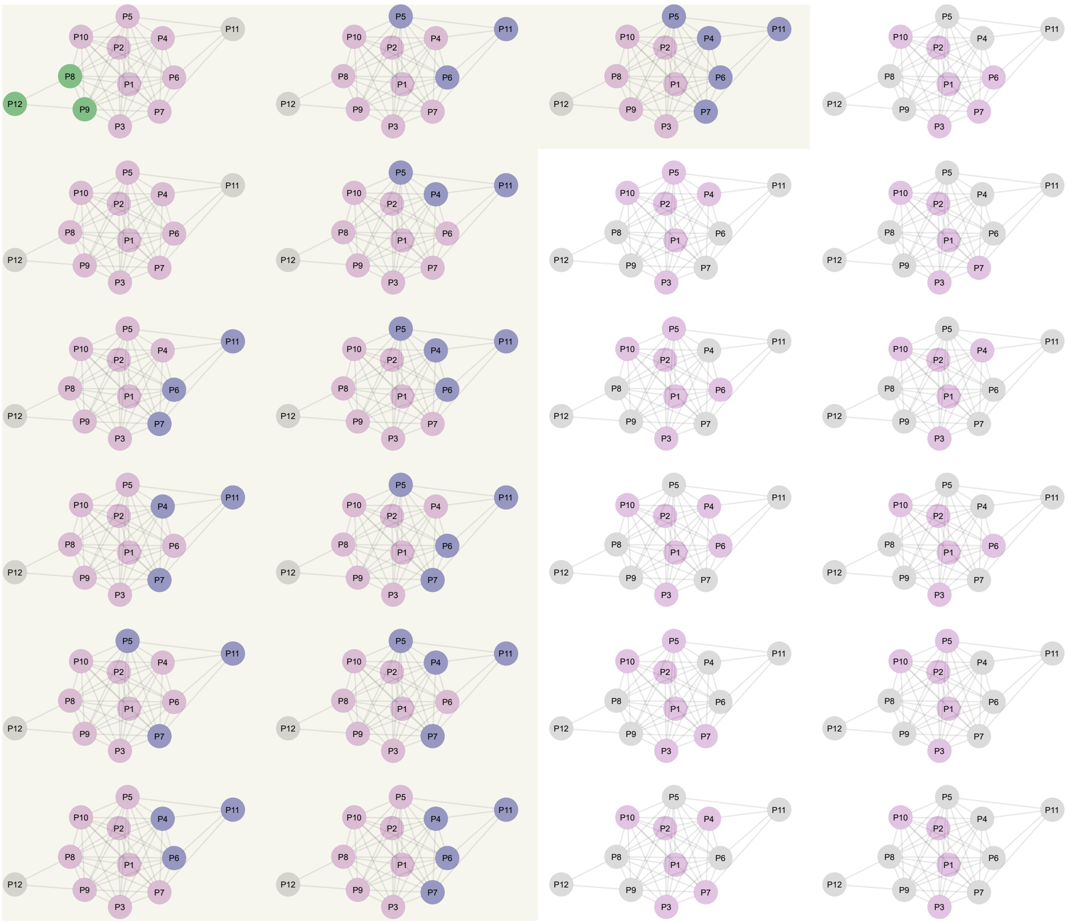

possible cliques for two seeds per iteration

additional possible cliques for three or more seeds per iteration

Figure 9: **All possible cliques that can be enumerated by ModuleDiscoverer using two seeds (highlighted yellow) or five seeds (all) per iteration.**

Table 1: **Probability for the identification of proteins that are members of only small cliques.** All possible cliques are grouped according to their mandatory set of protein members. Complementary clique members are outlined in braces. Probability of occurrence for all groups of cliques in 10,000 iterations is given for the single-seed approach (1 seed) as well as the multi-seed approach using two, three or four seed proteins per iteration.

cliques containing	1 seed	2 seeds	3 seeds	4 seeds
$P1, P2, P3, P10, \{P4, P5, P6, P7, P8, P9\}$	0.7696	0.6912	0.6426	0.6106
$P11, \{P4, P5, P6, P7\}$	0.1335	0.1906	0.2344	0.2597
$P8, P9, P12$	0.9690	0.1182	0.1229	0.1297

or $P1, P2, P3, P10$ for the single-seed approach and the multi-seed approach for two to four seeds per iteration. The results show that the multi-seed approach increases the probability for the identification of proteins that are part of small cliques only. Using the single-seed approach, such proteins could be missed due to the favored enumeration of large maximal cliques.

Results of the multi-seed approach

To observe the influence of the number of seeds on the identified regulatory module we applied ModuleDiscoverer using 1,000,000 iterations with either two or five seed nodes per iteration. Using two seed nodes a total of 1,926,551 cliques was identified. Of these cliques we found 108,143 unique cliques including all 1,341 maximal cliques. The five seed-node approach identified a total of 4,536,567 cliques, which enclose a total of 525,890 unique cliques. Four of the 1,341 maximal cliques were not found. This shows that, depending on the number of initial seed nodes used, the multi-seed approach is also able to identify all maximal cliques in a given PPI network.

Both of the identified regulatory modules are shown in figure 10. Since all maximal cliques were identified using two seeds per iteration, the resulting regulatory module (Figure 10, left) encloses the reference regulatory module shown in figure 4. Apart from the 60 nodes and 282 edges of the reference regulatory module, the two-seed based regulatory module contains 12 more proteins and 280 more edges. Five of these 12 proteins were measured on the microarray but not part of the 286 DEGs.
The identified regulatory module using five seed nodes (Figure 10, right) is

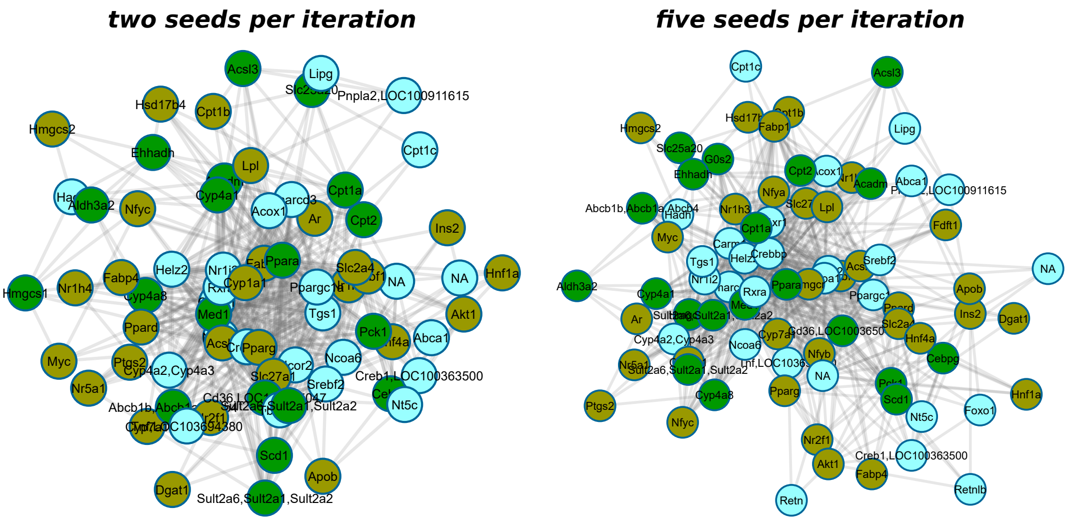

Figure 10: **The regulatory modules identified based on the multi-seed approach using 1,000,000 iterations with two seeds (left) and five seeds (right) per iteration.** The color of the nodes corresponds to proteins associated with a DEG (green), proteins associated with a gene that was measured in the experiment but not differentially expressed (brown) and proteins that are within the PPIN but not present on the microarray (blue). Nodes are labeled with the official gene symbol or 'NA', if the official gene symbol is not available.

composed of 80 proteins and 668 connecting edges. 50 of the 80 proteins are associated to genes that were measured on the microarray. 20 of these proteins are associated to DEGs. Notably, the regulatory module identified using five seeds per iteration (Figure 10, right) encloses both, the regulatory module using two seeds per iteration (Figure 10, left) as well as the reference regulatory module (Figure 4).

Performance of the multi-seed approach

We evaluated the performance of the multi-seed approach by computing the similarity between the reference regulatory module (Figure 4) and the identified regulatory module for 14 increasing numbers of iterations (Figure 11). Using two seeds per iteration (Figure 11, circles) we found that on average 40,000 iterations were required to recover the reference regulatory module. At least 100,000 iterations were required for recovery in 95% of 100 bootstrap samples. Using five seeds per iteration we found these numbers to be higher.

Here, the reference regulatory module was recovered at an average of 75,000 iterations, with an upper 95% bound of 150,000 iterations.
Regarding the stability of both regulatory modules, our results (Figure 11) show that complete stability was not reached within 1,000,000 iterations but are above 93% for both, nodes and edges.

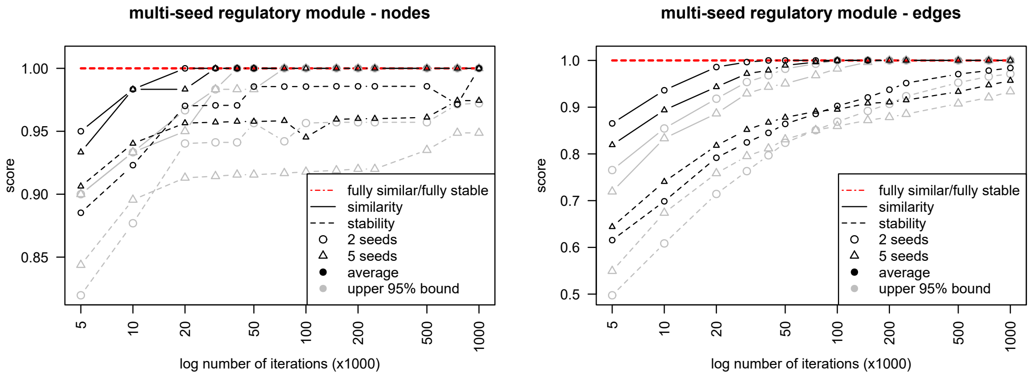

Figure 11: **Similarity of the identified regulatory modules using two seeds (top) and five seeds (bottom) per iteration to the reference regulatory module based on increasing number of iterations.**

1.7 Discussion

To test the performance of ModuleDiscoverer, we used a small *PPAR-alpha* -centered sub-network that encloses all first neighbors of the transcription factor *PPAR-alpha* in the high-confidence PPIN provided by the STRING database. Although the network is very small, it serves the purpose that (i) all maximal cliques in the network can be enumerated using *igraph*, (ii) the expression data by Baumgardener *et al.* [3] can be applied and (iii) the network reflects the property of highly overlapping cliques. However, in future studies it would be interesting to assess the performance of our algorithm using *in-silico* PPINs and expression data.

A motivation for the development of ModuleDiscoverer was our observation that the computational demanding enumeration of all maximal cliques in a PPIN is not necessary for the identification of regulatory modules. Using the *PPAR-alpha* -centered sub-network, we found that only 1,051 out of all 1341 maximal cliques, i.e., app. 78% of all maximal cliques, are potentially

relevant for the regulatory module. However, we found that this percentage of relevant cliques decreases when using the full high-confidence PPIN of *R. norvegicus*. Enumeration of maximal cliques in this PPIN using the single-seed approach of ModuleDiscoverer with 1,000,000 iterations identified 185,178 unique maximal cliques (see manuscript). 19,638 of these 185,178 maximal cliques (app. 9.43%) contained at least one of the 286 DEGs, i.e., were potentially relevant for the regulatory module. Since we do not know all maximal cliques, these numbers are estimates. However, they support the assumption that an approximation of the community structure underlying a PPIN can be sufficient for the identification of a biologically relevant regulatory module.

Using the single-seed approach, we showed that our algorithm can identify all maximal cliques in a given PPI network. Consequently, given a large enough number of iterations the identified regulatory modules will equal the 'true' regulatory modules. One of the main drawbacks of the single-seed approach is that in dense regions of highly overlapping cliques the identification of large cliques is favored. Consequently, given a fixed number of iterations the probability for proteins that are part of only small maximal cliques to contribute to the identified regulatory module is much lower compared to proteins that are members of large maximal cliques.
To tackle this problem, we introduced the multi-seed approach. We demonstrated that in dense regions of highly overlapping cliques the algorithm will shift from the identification of mainly large cliques towards the identification of smaller cliques. As a consequence, the probability for proteins that are members of small cliques only to contribute to the final regulatory module increases. A drawback of the multi-seed approach is that the breakdown of large maximal cliques into its smaller cliques can lead to the inflation of the regulatory module with proteins that are not associated to DEGs. To this end, after their identification, the regulatory modules should always be tested for their significant enrichment with proteins associated to DEGs. This ensures relevance of the identified modules. Furthermore, the drastic increase in possible cliques in the PPIN leads to higher numbers of iterations required to reach stability of the regulatory module.
An advantage of the multi-seed approach is its reduced computational cost. In each iteration, the multi-seed approach processes multiple cliques simultaneously. They compete for nodes in the PPIN, which can reduce the number of their potential neighbors during their extension. For the *PPAR-alpha* -centered sub-network, we found that already the use of two seeds per iteration decreases the time per clique to about 80% compared to the seconds per clique for the single-seed approach (Table 2).

Table 2: **Time required for the enumeration of each clique in reference to the single-seed approach.**

	1 seed	2 seeds	5 seeds
time per clique	100%	88.52%	68.15%

1.8 Conclusion

We have evaluated the performance of ModuleDiscoverer, an algorithm for the identification of regulatory modules based on large-scale whole-genome PPI networks in conjunction with gene expression data from high-throughput experiments. We demonstrated that ModuleDiscoverer can successfully recover reference regulatory modules using either the single-seed or the multi-seed approach. On the one hand, the single-seed approach is suitable for the identification of regulatory modules that are comparable to the results of current algorithms. On the other hand, the multi-seed approach complements the single-seed approach by recovering the single-seed-based module faster at the price of larger modules due to additional proteins. In that, regulatory modules based on the multi-seed approach can be seen as a comprehensive extension to the single-seed based regulatory modules. Independent of the approach used, validity of the identified regulatory module should always be assessed by (i) testing for significant enrichment of the regulatory module with proteins associated with DEGs and (ii) additional data such as single nucleotide polymorphism (SNP) data from independent genome-wide association studies associated with the condition represented by the experimental high-throughput expression data used.

2 Robustness of ModuleDiscoverer to noise in the PPIN

Using ModuleDiscoverer the identification of regulatory modules is dependent on the protein-protein interaction network (PPIN) used. Since the true underlying PPIN of an organism is generally unknown, network sources such as STRING provide a confidence score for each edge stating how certain the user can be about the respective edge. Apart from the recommendation of STRING to consider edges with a score above 0.7 as high-confidence edges,

the choice of an appropriate cutoff is left to the user. While choosing a low cutoff will increase the number of false edges (false positives), the choice of a high cutoff will exclude truly existing edges from the PPIN, i.e., increase the number of false negative edges. Ideally, one would calculate the regulatory module for each cutoff and subsequently select the best cutoff based on some score measuring the validity of the module. However, since identification of the regulatory modules for each cutoff is likely computationally infeasible, one has to decide for a single cutoff. Consequently, the question arises to which extend the regulatory modules identified by ModuleDiscoverer differ with varying edge score around the selected cutoff.

From a theoretical point of view, an increase (decrease) in the cutoff from 0.7, e.g., 0.75 (0.65), leads to the deletion (inclusion) of edges in the PPIN and thus, possibly to a decrease (increase) in the size of the identified cliques. Depending on the node deleted from (added to) a clique, there are three possible consequences. (i) The node is not part of the proteins associated with background genes, i.e., genes that are present on the microarray. In this case, the node is an exception node which will simply be deleted from (added to) the module if its clique is significantly enriched with proteins associated to differentially expressed genes (DEGs). (ii) The node is part of the proteins associated with background genes. This will lead to a decrease (increase) in the p-value of the clique and thus, possibly to the inclusion (exclusion) of the clique. (iii) The node is part of the proteins associated to DEGs leading to an increase (decrease) in the p-value of the clique. Consequently, the clique might be removed from (added to) the final module. These three possibilities of course depend on the assumption that the overall number of proteins present in at least one clique does not change significantly. In other words, whether or not a protein is part of a clique or not has to be independent of the selected cutoff for the edge score. This is important because ModuleDiscoverer uses the overall number of proteins that belong to at least one clique as background for the Fisher's exact test. The Fisher's exact test in turn is used to determine the enrichment of cliques with proteins associated to DEGs. If this background is altered, the change of the p-value does not depend anymore solely on the property of the altered node, i.e., whether or not the node is a protein associated to a background gene, a differentially expressed gene or none of the both. However, this should only be an issue for very high cutoffs of the edge score. This is because we can expect that due to the limited number of edges in such a high-cutoff PPIN the probability for a node to be part of a clique is much lower compared to the same node in a PPIN with plenty edges.

To test the robustness of ModuleDiscoverer towards changes in the selected cutoff, we re-identified the NASH-regulatory module based on the STRING

PPINs containing only edges with a score greater 0.65 and 0.75, respectively. For the single-seed and the multi-seed approach separately, we then compared these two modules with the NASH-regulatory module based on the respective PPIN with cutoff 0.7. The results show that for the single-seed (Figure 12, left) as well as the multi-seed approach (Figure 12, right) a conserved set of core proteins exists. For the single-seed approach these 236 proteins make up for 87.41 percent of all proteins in the 0.75-cutoff-based module, 75.90 percent of all proteins in the 0.7-cutoff-based module and 57.42 percent of all proteins in the 0.65-cutoff-based module. These numbers are similar for the regulatory modules identified using the multi-seed approach (331 proteins shared; 0.75-cutoff-based: 87.11 percent; 0.7-cutoff-based: 61.64 percent; 0.65-cutoff-based: 61.64 percent).

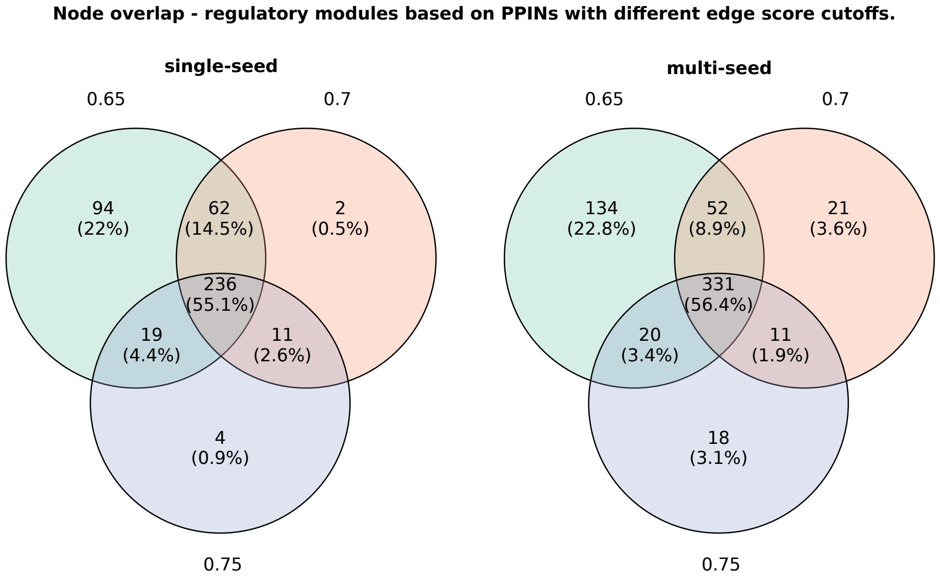

Figure 12: **Node overlap between regulatory modules identified based on PPINs with varying edge score.** Outlined are the regulatory modules using the single-seed approach (left) as well as the multi-seed approach (right).

Furthermore, we assessed similarity of the identified modules resulting from PPINs with varying edge score cutoffs based on similarity of their GeneOntology (GO)-enrichment results. Using the method outlined in the manuscript (see methods section in the manuscript; Comparing modules based on lists of GO-terms), our results (Figure 13) show that independent of the ontology used the ModuleDiscoverer-identified regulatory modules form a clade in the dendrogram. As such, they are more related to each other com-

pared to the modules derived from DEGAS, MATISSE, KeyPathwayMiner or the set of DEGs.

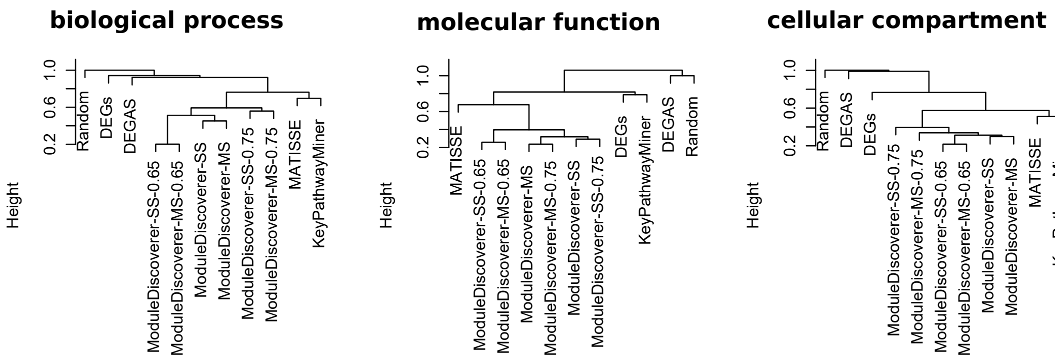

Figure 13: **Similarity of different regulatory modules based on results of GO enrichment tests.** Regulatory modules identified using DEGs, DEGAS, KeyPathwayMiner and Matisse are based on the STRING PPIN with an edge score > 0.7. For the regulatory modules identified using ModuleDiscoverer the edge score used as cutoff is outlined in the label.

Finally, we tested all identified modules for their enrichment with proteins associated to genes known to be affected by single nucleotide polymorphisms (SNPs) related to non-alcoholic fatty liver disease (NAFLD). We exploited for this analysis the information derived from the DisGeNet database (see methods section in the manuscript for details). The result (Table 3) shows that all regulatory modules identified by ModuleDiscoverer are significantly enriched (p-value < 0.05) for proteins corresponding to SNP-associated genes in NAFLD. Notably, when using ModuleDiscoverer the p-value is decreasing with increasing cutoff of the edge score.

In conclusion, this analysis demonstrates that with decreasing (increasing) edge cutoff the core set of proteins in the regulatory module is gradually extended (diminished), independent of the number of seeds used. Thus, we can consider the regulatory modules identified by ModuleDiscoverer as robust to noise in the network.

Table 3: **Enrichment of the different identified regulatory modules with proteins associated to SNP-affected genes relevant for NAFLD.**

Algorithm	Approach	Edge score cutoff	P-value
DEGs	-	-	2.99E-01
DEGAS	-	0.70	1.00E+00
MATISSE	-	0.70	1.11E-01
KeyPathwayMiner	-	0.70	1.81E-01
ModuleDiscoverer	single-seed	0.65	4.77E-03
ModuleDiscoverer	single-seed	0.70	4.24E-04
ModuleDiscoverer	single-seed	0.75	1.39E-04
ModuleDiscoverer	multi-seed	0.65	1.06E-02
ModuleDiscoverer	multi-seed	0.70	1.23E-03
ModuleDiscoverer	multi-seed	0.75	4.35E-04

References

[1] Barrenäs, F. *et al.* Highly interconnected genes in disease-specific networks are enriched for disease-associated polymorphisms. *Genome Biol.*, **13,** R46 (2012)

[2] Eblen, J., Phillips, C. A., Rogers, G. L., Langston, M. A. The maximum clique enumeration problem: algorithms, applications, and implementations. *BMC Bioinformatics*, **13,** 1 (2012)

[3] Baumgardner, J. N., Shankar, K., Hennings, L., Badger, T. M., Ronis, M. J. A new model for nonalcoholic steatohepatitis in the rat utilizing total enteral nutrition to overfeed a high-polyunsaturated fat diet. *Am J Physiol Gastrointest Liver Physiol.*, **294,** G27–G38 (2008)

[4] Szklarczyk, D. *et al.* String v10: protein-protein interaction networks, integrated over the tree of life. *Nucleic Acids Res.*, **43,** D447–D452 (2015)

[5] Pawlak, M., Lefebvre, P., Staels, B. Molecular mechanisms of pparα action and its impact on lipid metabolism, inflammation and fibrosis in non-alcoholic fatty liver liver disease. *Journal of hepatology*, **62,** 720–733, (2015)

[6] Csardi, G., Nepusz, T. The igraph software package for complex network research. *InterJournal.* **Complex Systems,** 1695, (2006)

[7] R Core Team R: A Language and Environment for Statistical Computing. *R Foundation for Statistical Computing, Vienna, Austria*, (2015)

3 Supplementary Figures

3.1 Supplementary Figure F1

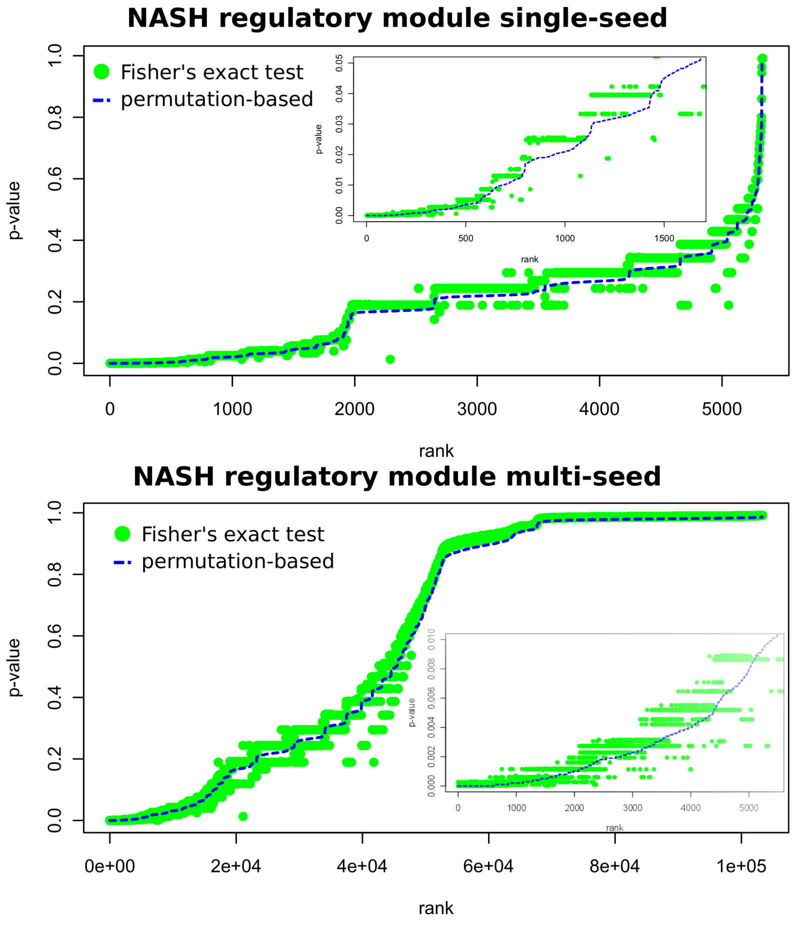

Figure F1: **Comparing permutation-based p-values with p-values calculated using Fisher's exact test.** Fisher-exact test (green dot) and permutation based (blue line) p-value for each clique over its rank of the permutation based p-value. Top: plot for single-seed regulatory module; Bottom: plot for multi-seed regulatory module.

3.2 Supplementary Figure F2

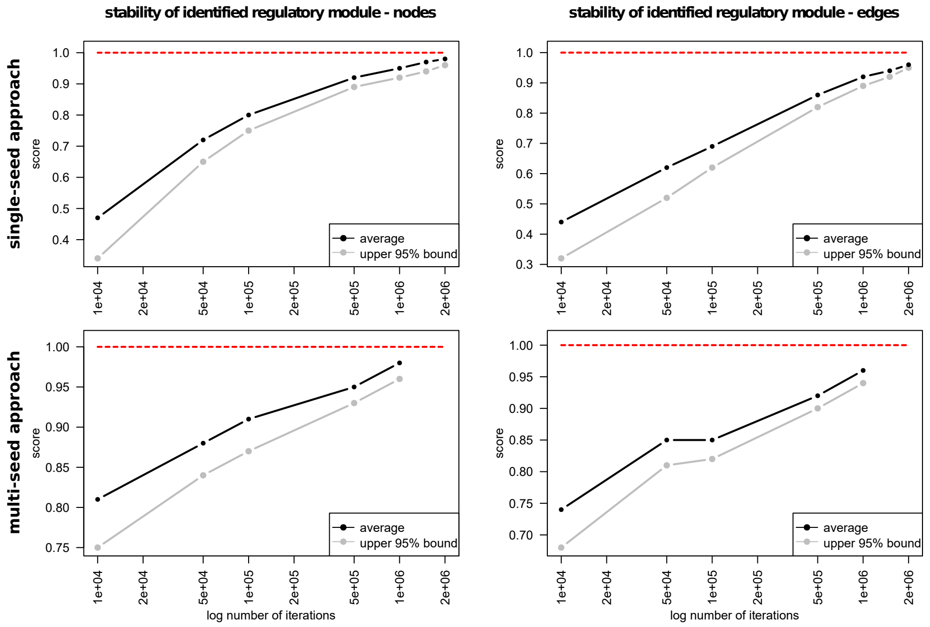

Figure F2: **Stability of the identified regulatory modules.** Plot outlining the stability in terms of nodes (left) and edges (right) of the single-seed (top) and the multi-seed (down) regulatory module for increasing numbers of iterations. Both plots show that with more iterations the stability of the identified regulatory modules increases. In other words, the use of additional iterations (and therefore the enumeration of additional cliques) has a negligible effect on the structure of the identified regulatory modules.
